# Structural and Functional Roles of Glycosylation in Fungal Laccase from *Lentinus* sp.

**DOI:** 10.1371/journal.pone.0120601

**Published:** 2015-04-07

**Authors:** Manuel Maestre-Reyna, Wei-Chun Liu, Wen-Yih Jeng, Cheng-Chung Lee, Chih-An Hsu, Tuan-Nan Wen, Andrew H.-J. Wang, Lie-Fen Shyur

**Affiliations:** 1 Institute of Biological Chemistry, Academia Sinica, Taipei, Taiwan; 2 Agricultural Biotechnology Research Center, Academia Sinica, Taipei, Taiwan; 3 Center for Bioscience and Biotechnology, National Cheng Kung University, Tainan, Taiwan; 4 Core Facilities for Protein Structural Analysis, Academia Sinica, Taipei, Taiwan; 5 Institute of Plant and Microbial Biology, Academia Sinica, Taipei, Taiwan; 6 Ph.D. Program for Translational Medicine, College of Medical Science and Technology, Taipei Medical University, Taipei, Taiwan; 7 Ph.D. Program for Translational Medicine, Kaohsiung Medical University, Kaohsiung, Taiwan; Instituto de Tecnologica Química e Biológica, UNL, PORTUGAL

## Abstract

Laccases are multi-copper oxidases that catalyze the oxidation of various organic and inorganic compounds by reducing O_2_ to water. Here we report the crystal structure at 1.8 Å resolution of a native laccase (designated nLcc4) isolated from a white-rot fungus *Lentinus* sp. nLcc4 is composed of three cupredoxin-like domains D1-D3 each folded into a Greek key *β*-barrel topology. T1 and T2/T3 copper binding sites and three *N*-glycosylated sites at Asn^75^, Asn^238^, and Asn^458^ were elucidated. Initial rate kinetic analysis revealed that the *k*_cat_, *K*_*m*_, and *k*_cat_/*K*_*m*_ of nLcc4 with substrate ABTS were 3,382 *s* ^-1^, 65.0 ± 6.5 μM, and 52 *s* ^-1^μM^-1^, respectively; and the values with lignosulfonic acid determined using isothermal titration calorimetry were 0.234 *s* ^-1^, 56.7 ± 3.2 μM, and 0.004 *s* ^-1^μM^-1^, respectively. Endo H-deglycosylated nLcc4 (dLcc4), with only one GlcNAc residue remaining at each of the three *N*-glycosylation sites in the enzyme, exhibited similar kinetic efficiency and thermal stability to that of nLcc4. The isolated *Lcc4* gene contains an open reading frame of 1563 bp with a deduced polypeptide of 521 amino acid residues including a predicted signaling peptide of 21 residues at the *N*-terminus. Recombinant wild-type Lcc4 and mutant enzymes N75D, N238D and N458D were expressed in *Pichia pastoris* cells to evaluate the effect on enzyme activity by single glycosylation site deficiency. The mutant enzymes secreted in the cultural media of *P*. *pastoris* cells were observed to maintain only 4-50% of the activity of the wild-type laccase. Molecular dynamics simulations analyses of various states of (de-)glycosylation in nLcc support the kinetic results and suggest that the local H-bond networks between the domain connecting loop D2-D3 and the glycan moieties play a crucial role in the laccase activity. This study provides new insights into the role of glycosylation in the structure and function of a Basidiomycete fungal laccase.

## Introduction

Laccases (para-diphenol: oxygen oxidoreductases, EC 1.10.3.2) are a family of multi-copper oxidases that catalyze the oxidation of a broad range of organic substrates such as polyphenols, diamines, and some inorganic compounds [1]. Due to their wide range of substrate affinities, laccases and/or laccase-mediator systems are exploited in various industrial processes, such as pulp paper bleaching, textile decolorization, bioremediation of soils and water, and pretreatment of lignocellulosics for bioethanol production [2]. Laccases are also applied in the production of new antibiotics derivatives and the synthesis of complex natural products. As they utilize atmospheric oxygen and produce water as the only by-product, they are more eco-friendly than traditional organic syntheses [3,4]. Laccases are widely distributed across two fungal phyla, Basidiomycetes and Ascomycetes, and some have been discovered from higher plants, bacteria and insects [5]. Fungal laccases usually consist of three cupredoxin-like domains (designated D1-D3) each of which folds into a Greek key β-barrel topology; four copper ions are located in three distinct types of metal binding sites (designated as T1-T3) in the enzymes [6,7]. The mononuclear T1 (blue copper) characterized by a strong absorbance at ~600 nm is located in D3 and the tri-nuclear cluster is formed by mononuclear T2 (normal copper, weakly absorbing) and binuclear T3 (absorbing at 330 nm) at the interface between D1 and D3 [6,8]. It is known that T1 (Cu1) is the primary electron acceptor site and also serves as the rate-limiting step in the catalytic process. T2 and the pair of T3 copper ions (Cu2 and Cu3) forming the trinuclear cluster also function as electron acceptors [5]. In addition, most fungal laccases are characterized as monomeric glycoproteins with molecular masses of 55–85 kDa. In general, the carbohydrate content in secreted laccases can be up to 25% with 3–10 potential glycosylation sties predicted based on the consensus amino acid sequence Asn-X-Thr/Ser [9]. To date, 16 non-redundant laccase crystal structures from 15 fungal strains have been deposited in the RCSB Protein Data Bank (http://www.rcsb.org/pdb/home/home.do). The information provided based on these fungal laccases crystal structures focuses mainly on the architecture of the substrate binding pocket and the organization of the loops that surround the four copper ions that are essential for catalytic activity in the active site [10]. Modifications of amino acid residues around the copper sites that manipulate the redox potentials of laccases have also been reviewed [11]. The glycosylation profile and its effect on enzymatic activity of laccase from a thermostable strain *Pycnoporus sanguineus* was reported [12]. De-glycosylation was observed to cause negative effect on laccase activity and stability mostly at lower temperature (20~30°C). The stability mechanisms in terms of ionic strengths, temperature, and glycosylation status in a thermophilic laccase were studied using molecular dynamics [13]. Nevertheless, the structure-function relationship of the glycan moieties in fungal laccases has not yet been fully investigated.

In an attempt to address the role of glycosylation in fungal laccases, we solved the crystal structure of a native laccase (nLcc4) purified from a newly identified Basidiomycete *Lentinus* sp. Three putative *N*-glycosylated sites were confirmed, among which two sites with high mannose structures were determined. The importance of the three *N*-glycosylation sites in the laccase were investigated by enzymatic de-glycosylation, site-directed mutagenesis, and molecular dynamic simulations of different glycosylation states of the protein. This is the first study to provide insight into the structure-function relationship of the glycan moieties in fungal laccase using an array of kinetic parameters and biochemical/physio-chemical properties observed in native and de-glycosylated laccases, in combination with the crystal structure and molecular dynamics simulations results. This study demonstrates that the first *N*-GlcNAc sugar in the three glycosylated sites plays an important role in stabilizing the structure of fungal laccase.

## Materials and Methods

### Chemicals

2,2'-Azino-bis(3-ethylbenzthiazoline-6-sulphonic acid) (ABTS), 2,6-dimethylphenol (2,6-DMP), lignosulfonic acid sodium salt, methanol, and 2,5-dimethylaniline were obtained from Sigma. Endoglycosidase H (Endo H), Endo H fused with maltose binding protein (Endo Hf), and peptide-*N*-glycosidase F (PNGase F) were from New England BioLabs. All chemicals were reagent grade or equivalent, unless otherwise stated.

### Purification of native laccase protein from *Lentinus* sp.

The target white-rot fungal strain, *Lentinus* sp. was identified by our group and used for laccase production. Purification followed a previously published method [2]. Briefly, *Lentinus sp*. was cultivated in a medium containing 2.4% potato dextrose broth, 5% soytone, and 0.4 mM CuSO_4_ at 25°C and 150 rpm for 18 days. The culture supernatant containing the laccase enzyme was concentrated using Labscale TFF System (Millipore) with a 10K Pellicon-XL filter, and dialyzed against 50 mM sodium phosphate buffer (pH 6.0). The concentrated crude laccase proteins were further purified on two sequential Q Sepharose ion exchange columns (2.6 × 30 cm) and a Superdex 200 gel filtration column (1.6 × 90 cm) to give the purified native laccase (designated nLcc4).

### Cloning of the lcc4 gene

Total RNA of *Lentinus* sp. was extracted and purified by TRIzol reagent (Invitrogen) according to the manufacturer’s instructions with minor modifications. The cDNA transcripts were obtained by reverse transcriptase polymerase chain reaction (RT-PCR) using Transcriptor First Strand cDNA Synthesis Kit (Roche), following another PCR to amplify the target gene, *Lentinus* sp. *lcc4*. The anchored oligo(dT)_18_ primer provided in the kit and the specific primers lcc4F and lcc4R listed in Table 1 were used in the PCR reaction. PCR amplifications were performed using the conditions: 94°C for 1 min, 30 cycles of 98°C for 10 s, 65°C for 2 min, and 68°C for 1 min, with a final extension at 68°C for 5 min. The amplified DNA fragments (1500 bp) were subcloned into the PCR-Blunt II-TOPO vector using Zero Blunt TOPO PCR Cloning Kit (Invitrogen) and confirmed by DNA sequencing.

**Table 1:** Nucleotide sequences of PCR primers.

Primer	Oligonucleotide sequence
lcc4F	5′-TTTTATGAAGGCCCTCTCCTTCCTTACACC-3′
lcc4R	5′-TTTTTCACTGGTCGTCCTCAGCTAGG-3′
lcc4F_no sp	5′-TTTGAATTCACTCCGTCGGGCCCGTAGCCAACCTCAAGATCG-3′
lcc4R_no sp	5′-TTTGCGGCCGCTCACTGGTCGTCCTCAGCTAGGGCGTCGTAG-3′
N75D_F	5′-CGATGAGCT**GAC**GGACCACACTATGCTC-3′
N75D_R	5′-GAGCATAGTGTG**GTC**CGTCAGCTCATCG-3′
N162D_F	5′-GTATGATGTTGAT**GAC**GAATCTACGGTGATC-3′
N162D_R	5′-GATCACCGTAGATTC**GTC**ATCAACATCATAC-3′
N238D_F	5′-CAGCATCGATGGTCAC**GAT**ATGACCATTATC-3′
N238D_R	5′-GATAATGGTCAT**ATC**GTGACCATCGATGCTG-3′
N458D_F	5′-GCGGCCGGCGAC**GAC**GTGACGATCCGTT-3′
N458D_R	5′-AACGGATCGTCAC**GTC**GTCGCCGGCCGC-3′

### LC-MS/MS glycosylation site analysis

To analyze the protein glycosylation sites using LC-MS/MS, the purified nLcc4 was either digested in-gel or in-solution using trypsin or Asp-N (Promega), and the digested peptides were further subjected to de-glycosylation using PNGase F (Promega) in the presence of ^18^O water (^18^O, 97%, CIL). The protease digested and de-glycosylated peptides were acidified in 0.1% TFA and desalted with Oasis HLB cartridge (Waters) for LC-MS/MS analysis. Meanwhile, Endo H-deglycosylated proteins were also subjected to protease (trypsin or Asp-N) digestion, and the digested peptides were analyzed on LC-MS/MS. An LC-nESI Q Exactive mass spectrometer (Thermo Fisher Scientific) coupled with an on-line nanoUHPLC (Dionex UltiMate 3000 Binary RSLCnano) was utilized for peptide glycosylation site analysis. An Acclaim PepMap 100 C18 trap column (75 μm × 2.0 cm, 3 μm, 100 Å, Thermo Scientific) and an Acclaim PepMap RSLC C18 nano LC column (75 μm × 15 cm, 2 μm, 100 Å) were used to deliver the solvent and separate peptides with a linear gradient from 3 to 30% of acetonitrile in 0.1% (v/v) formic acid for 90 min at a flow rate of 300 nL/min. The acquisition of the MS data was performed in data dependent mode with a full MS scan followed by 10 MS/MS scans of the top 10 precursor ions from the MS scan. The MS scan was performed with a resolving power of 70,000 over the mass-to-charge (*m/z*) range 380 to 1800 and dynamic exclusion enabled. The data dependent MS/MS acquisitions were performed with the parameters: 2 *m/z* isolation window, 27 NCE, and 17,500 resolving power. Peptide glycosylation site mapping was performed using the Proteome Discoverer software (v.1.4, Thermo Fisher Scientific) with SEQUEST and Mascot (v.2.4, Matrix Science) search engines against a laccase protein database with 11,740 sequence entries downloaded from NCBI plus the amino acid sequence of nLcc4 derived from its cDNA identified from this study. The parameters for database searches were set as follows: full trypsin or Asp-N digestion with 2 maximum missed cleavage sites, precursor mass tolerance = 10 ppm, fragment mass tolerance = 0.02 Da; dynamic modifications: oxidation (M), and deamidation with one isotopic ^18^O substitution (PNGase F de-glycosylation) or HexNAc (Endo H de-glycosylation) modification (N); static modifications: carbamidomethyl (C). The peptide-spectrum matches (PSM) were validated using fixed score thresholds for each search engine, and q-values and posterior error probabilities by Percolator algorithm against the decoy database search. All the peptides were filtered with a *q*-value threshold of 0.01 (1% false discovery rate), and Mascot significant threshold *p* ≤ 0.05 or Sequest XC or score thresholds (*vs*. charge state): ≥ 2.0 (+2), 2.25 (+3), 2.5 (+4).

### Construction of plasmids of wild-type and lcc4 mutants for recombinant protein expression in *P*. *pastoris*

The *lcc4* gene with the deletion of a putative signal peptide sequence was amplified using primers lcc4F_no sp and lcc4R_no sp as listed in Table 1. PCR amplifications were performed as described above. The amplified DNA fragments were digested with *EcoR*I and *Not*I and then subcloned into pPICZα-B vector (Invitrogen) to produce plcc4-WT. Four mutant forms of *lcc4* genes with Asn→Asp mutation at positions Asn^75^, Asn^162^, Asn^238^, and Asn^458^ were prepared in plcc4-WT gene plasmid using the QuikChange Lightning Site-Directed Mutagenesis Kit (Agilent) to produce N75D, N162D, N238D, and N458D. The primers used for mutagenesis with the base changes from the wild-type gene sequence (underlined) are listed in Table 1.

### Expression of recombinant lcc proteins in *P*. *pastoris*

*P*. *pastoris* X33 host cells carrying plcc4-WT, N75D, N162D, N238D, and N458D, respectively were grown according to the manufacturer’s instructions (Invitrogen). Briefly, sterile BMMH media (500 mL in a 2 L baffled shake flask) containing 0.4 mM CuSO_4_ and Zeocin (100 μg/mL, Invitrogen) was inoculated with 2% overnight culture (OD_600_ = 2) and grown at 30°C for 13 days at 110 rpm in a shaking incubator. The protein expression was induced by 100% methanol (final 0.5% v/v) every 24 h. The laccase proteins produced and secreted in the supernatant were monitored daily by standard laccase activity assay, and the protein concentration was determined using the Bradford assay (Bio-Rad) with bovine serum albumin as a standard.

### Kinetic studies

Laccase activity was determined as described previously with minor modifications [2,14]. The optimal temperature for laccase activity was determined using standard laccase activity assay with various incubation temperatures (20–90°C). The optimal pH for laccase activity was investigated at pH 2.0–8.0. The buffer systems used in this study were (50 mM for each): glycine-HCl buffer (pH 2.0–2.5), citrate buffer (pH 2.5–5.0), and sodium phosphate buffer (pH 6.0–8.0). The kinetic parameters *k*_cat_ and *K*_*m*_ were determined on different substrates in a 50 mM citrate buffer at the optimum pH and temperature, i.e., 0–1 mM for ABTS in a 50 mM citrate buffer (pH 2.5) at 70°C, and 0–2 mM for 2,6-DMP in a 50 mM citrate buffer (pH 3.5) at 65°C. The reaction was initiated by adding the enzyme. The oxidation rates of ABTS and 2,6-DMP were spectrophotometrically measured at 420 nm (Σ = 36,000 M^**−**1^cm^**−**1^) and 468 nm (Σ = 49,600 M^**−**1^cm^**−**1^), respectively. The kinetic parameters were obtained by non-linear regression to the Michaelis-Menten equation using software EnzFitter (Biosoft). One unit of enzyme activity was defined as the amount of enzyme that oxidized 1 μmol of substrate per minute.

### Determination of nLcc4 kinetics for lignosulfonic acid using isothermal titration calorimetry

Isothermal titration calorimetry (ITC) analysis was performed using an iTC-200 instrument (MicroCal, Piscataway, NJ) equipped with a sample cell (200 μL), a reference cell (200 μL) and an automatic injection syringe (40 μL). The apparent enthalpy (Δ*H*_app_, cal/mol) and the enzyme reaction rate were determined by single and multiple injection methods, respectively [15]. All the samples and substrates were prepared in 50 mM citrate buffer, pH 3.0. For the single injection method, the reference cell was filled with 200 μL deionized water, and 5 μL of 0.1 mM lignosulfonic acid solution was injected two times into the sample cell containing 200 μL of 1.39 μM nLcc4 solution with 1000 sec spacing between injections. The thermal power was recorded every 5 s with stirring speed 1000 rpm at 30°C in all assays. For the multiple injection method, 200 μL of nLcc4 solution was placed in the sample cell and 1 mM lignosulfonic acid solution was injected successively by 30 injections of 1 μL with 60 s spacing between injections.

Δ*H*_app_ refers to the amount of heat consumed or released from 1 mole of substrate completely converted to product and was calculated by Eq 1,

$$\Delta H_{app}=\frac{1}{{\left[S\right]}_{total}\cdot V}\int \frac{dQ(t)}{dt}dt$$ (1)

where [*S*]_*total*_ is the molar concentration of substrate completely converted to product, *V* is the volume of solution in the sample cell, and *dQ*/*dt* is the thermal power.

The enzyme reaction rate was determined by Eq 2,

$$Rate=\frac{d\left[P\right]}{dt}=\frac{\text{1}}{V\cdot \Delta H_{app}}\cdot \frac{dQ}{dt}$$ (2)

where Δ*H*_app_ calculated from Eq 1 was applied in Eq 2 to obtain the enzyme reaction rate.

The residual substrate concentration [*S*]_*t*_ as a function of time was determined by Eq 3,

$${\left[S\right]}_{t}={\left[S\right]}_{total}-{\left[P\right]}_{t}={\left[S\right]}_{total}-\frac{\int \frac{dQ}{dt}dt}{\Delta H_{app}\cdot V}$$ (3)

where [*P*]_*t*_ is the concentration of substrate converted to product at time *t*.

According to Eqs 1–3, the enzyme reaction rate and residual substrate concentration as a function of time were calculated to fit the Michaelis-Menten equation to obtain the kinetic parameters *k*_cat_ and *K*_*m*_.

### Deglycosylation reaction

The deglycosylation reaction of purified native laccase using Endo Hf (or Endo H) was carried out according to the manufacturer’s instructions. Briefly, 12.2 ng of pure native lcc4 was incubated with one unit of Endo H or Endo Hf at 37°C overnight to generate dLcc4 protein. After completion of the de-glycosylation reaction, the product derived by Endo Hf digestion was further purified using amylase magnetic beads (NEB) to remove Endo Hf, and the purity of dLcc4 was then confirmed by 10% SDS-PAGE. The dLcc4 derived from Endo H digestion was subjected to molecular mass and zymography analyses, and the further purified dLcc4 after Endo Hf digestion was used in the kinetic study.

### Crystallization and data collection

The crystals of laccase were grown using the sitting-drop vapor diffusion method. Protein droplets were prepared by mixing 0.2 μL protein solution and 0.2 μL reservoir solution using a robotic crystallization system (Phoenix RE, Rigaku), and equilibrated at 298 K against 50 μL reservoir solution. The crystal with a *P*2_1_2_1_2 space group was obtained using a reservoir solution consisting of 20% isopropanol, 20% polyethylene glycol 4000, and 100 mM sodium citrate, pH 5.6. The laccase crystal was flash-cooled to 100 K in a stream of cold nitrogen prior to data collection. Prior to flash cooling, the crystal was covered by perfluoropolyether (PFO-X175/08, Hampton Research) to avoid isopropanol evaporation and subsequently soaked briefly in a cryoprotectant solution containing 20% isopropanol, 20% polyethylene glycol 4000, and 20% glycerol in 100 mM sodium citrate buffer, pH 5.6. The diffraction data were collected from a single crystal using a Rayonix MX225HE CCD detector at the wavelength of 0.90000 Å on the BL44XU beamline (SPring-8, Japan). All data were processed and scaled using the HKL2000 program package [16].

### Structure determination and refinement

nLcc4 protein structure was determined by molecular replacement with Phaser [17] using the laccase structure from the white-rot fungus *Trametes versicolor* (PDB #1KYA) [18] as a search model. Automatic model building was performed with ARP/wARP [19] and Buccaneer software [20]. Model completion and refinement were performed with Refmacs [21] and Coot [22]. A subset of 5% randomly selected reflections was excluded from computational refinement to calculate the *R*_free_ factor throughout the refinement [23]. The refinements were carried out using Refmac 5 with TLS group tensor and anisotropic B factor without NCS restrains. The stereochemistry and structure of the final models were analyzed by Rampage [24] of the CCP4 program suite [25]. Data collection and refinement statistics are summarized in Table 2, and the atomic coordinates and structure factors have been deposited in the Protein Data Bank with an accession code 3X1B. The structural figures were produced using PyMOL (DeLano Scientific, http://www.pymol.org) and UCSF Chimera [26].

**Table 2:** Data collection and refinement statistics of nLcc4.

Protein Data Bank code	3X1B
Data collection	
Radiation source	SPring-8 BL44XU
Wavelength (Å)	0.900
Space group	*P*2_1_2_1_2
Unit cell parameter	
a (Å)	99.27
b (Å)	188.68
c (Å)	65.41
Resolution (Å)	30–1.80 (1.86–1.80)^a^
Number of reflections	114,514 (11,262)
Completeness (%)	99.8 (99.5)
Redundancy	6.4 (5.9)
Rmerge (%)	8.5 (58.4)
I/σ (I)	21.8 (2.6)
Overall Wilson B factor (Å^2^)	27.7
Refinement	
Resolution (Å)	30–1.80 (1.90–1.80)
Reflections (work)	108,673 (15,524)
Reflections (free)	5,728 (857)
*R*_work_ (%)	14.5 (19.0)
*R*_free_ (%)	19.7 (26.2)
Geometry deviations	
Bond length (Å)	0.007
Bond angles (o)	1.4
Mean B-values (Å^2^) / No.	
Protein atoms	27.1 / 7628
Carbohydrate atoms	48.9 / 327
Ion atoms	26.5 / 8
Water molecules	40.4 / 1,116
Ramachandran plot (%)^b^	
Favored	98.4
Allowed	1.2
Disallowed	0.4

^a^ Values in the parentheses are for the highest resolution shell.

^b^ Categories were defined by RAMPAGE

### Docking and molecular dynamics simulations

Suitable binding pockets were first approximated by performing a structure based alignment, which were then docked with a lignosulfonic acid monomer using Vina [27]. PyMOL (DeLano Scientific, http://www.pymol.org) and Autodock/Vina plugins were used to determine grid sizes and visualize ligand poses. Antechamber software and the General Amber Force Field (GAFF) were used for ligand parameterization, with total charges added manually, and partial charges calculated by Antechamber [28]. Glycan parameterization was based on GLYCAM06 [29] templates.

The single and tri-nuclear copper clusters in the laccase binding pocket were parameterized by the Metal Center Parameter Builder (MCPB) included in the AmberTools 13 package [30], supported by quantum mechanical calculations with the Gaussian 09 software package. All coppers were modeled as Cu(II), which is the only species compatible with the geometry of the binding pocket in our crystal structure.

MD simulations were carried out using Amber12. For the 20 ns simulations (2 ns for equilibration and 18 ns of production MD), the glycoprotein model based on the 3-D structure of nLcc4 (PDB #3X1B) was surrounded with a 10 Å TIP3PBOX water box. The glycoprotein was then subjected to energy minimization for 5000 cycles with 500 kcal/mol ^**−**^ Å^2^ harmonic restraints, followed by 5000 cycles where the restraints only applied to the protein component. Finally, the system was subjected to 5000 cycles of restraint free steepest descent minimization. The system was then equilibrated for 100 ps to 303 K using a randomly seeded Langevin thermostat (γ = 5 ps^**−**1^), and weak 10 kcal/mol ^**−**^ Å^2^ restraints, with Shake [31] constrained bond lengths for hydrogens. In the final step, constant pressure was introduced by slowly raising the system pressure to one atmosphere for further 50 ps at 303 K, with all other values left as before. The resulting models were then treated following guidelines reported previously in the literature for the treatment of protein-glycan complexes and glycoproteins [32].

Trajectories were evaluated with visual molecular dynamics (VMD) [33,34], which was also used for movie rendering, while ptraj was used for wrapping and hydrogen bond analysis. Theoretical ligand binding energies were calculated using the MMPBSA.py [35] script contained in the AmberTools 13 package. RMSD and simulated B-factors were plotted with Qtiplot (http://soft.proindependent.com/qtiplot.html).

## Results

### Characterization of nLcc4 protein and gene

**Fig 1:**
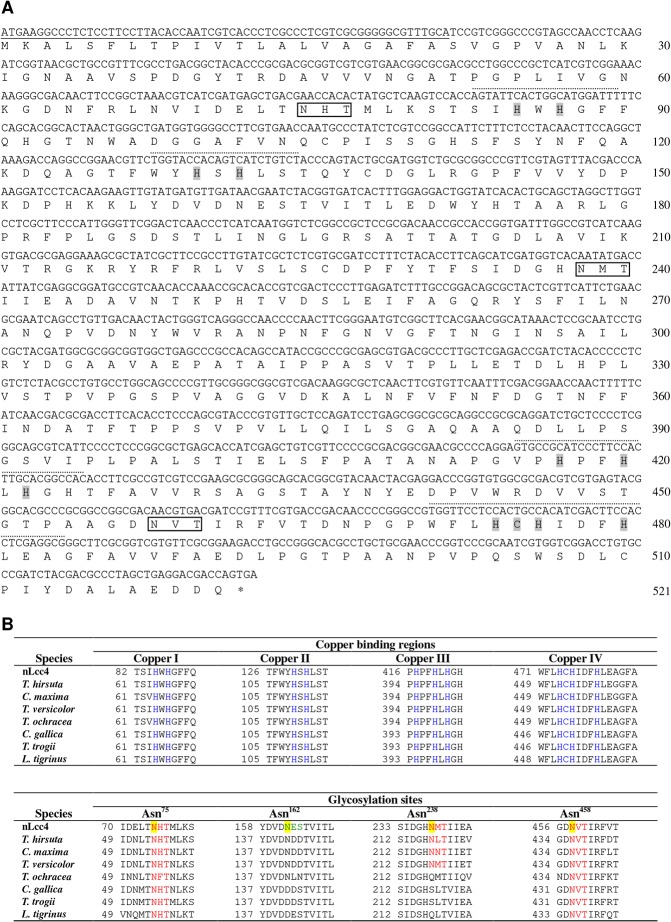
(A) The cDNA and deduced amino acid sequences of nlcc4. (B) The sequence alignments of *Lentinus* sp. nlcc4 with other Basidiomycete laccases. The signal peptide and the predicted four Cu binding regions are indicated by solid and dotted lines, respectively. The confirmed *N*-glycosylation sites (Asn-X-Ser/Thr) based on the nLcc4 sequence are framed or labeled in red. The predicted *N*-glycosylation site on nLcc4 is labeled in green. Residues coordinated with coppers are highlighted in grey or labeled in blue. Residues examined by point mutation (N→D) for production of recombinant mutants are highlighted in yellow. nLcc4 (PDB #3X1B), *Trametes hirsute* (PDB #3FPX), *Cerrena maxima* (PDB #3DIV), *Trametes versicolor* (PDB #1KYA), *Trametes ochracea* (PDB #2HZH), *Coriolopsis gallica* (PDB #4A2E), *Trametes trogii* (PDB #2HRG), and *Lentinus tigrinus* (PDB #2QT6).

An extracellularly secreted laccase, designated nLcc4 expressed by white-rot fungus *Lentinus* sp. was purified from an 18-day culture with homogeneity > 96% as judged by 10% SDS-PAGE. nLcc4 was determined to be a glycoprotein by periodic acid-Schiff staining (data not shown), and the carbohydrate content was determined to be approximately 6.5% by comparison of the molecular weight of the native and PNGase F-deglycosylated proteins by SDS-PAGE. The full-length *lcc4* gene was cloned using RT-PCR and deposited in GenBank with accession number KF836751. The *lcc4* gene contains an open reading frame of 1563 bp with a deduced polypeptide of 521 amino acid residues including a predicted signaling peptide of 21 amino acid residues at the *N*-terminus (Fig 1A). Four copper ion binding regions were identified in the lcc4 primary sequence which had almost 100% sequence identity to other Basidiomycete laccase sequences (Fig 1B); four potential glycosylation sites were observed at residues Asn^75^, Asn^162^, Asn^238^, and Asn^458^ based on the consensus amino acid sequence Asn-X-Thr/Ser. Asn^75^ and Asn^458^ are highly conserved among laccase proteins; partial homology was found for residue Asn^238^, whereas no identity was found for Asn^162^. We thus further carried out PNGase F-deglycosylation of nLcc4 and then subjected native and de-glycosylated proteins to trypsin digestion and tandem mass spectrometric analysis. By referring to the deduced primary amino acid sequence of *nlcc* cDNA, our MS results confirmed that Asn^75^, Asn^238^ and Asn^458^ are the *N*-glycosylation sites in nLcc4 (Table A in S1 File).

### Endo H deglycosylation of nLcc4

**Fig 2:**
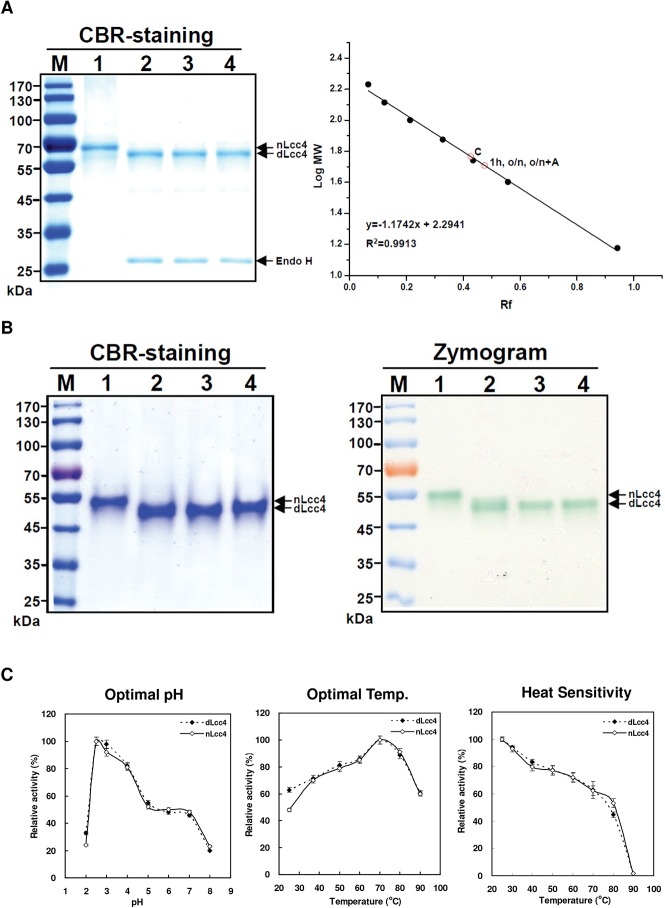
Analysis of molecular weight and enzymatic activity of native and Endo H-deglycosylated Lcc4 by 10% SDS-PAGE and zymography. (A) Heated laccases were separated and visualized by coomassie brilliant blue (CBR) staining and their relative mobility distances (R_f_ values) and molecular weights were calculated and calibrated with a set of molecular weight standards. (B) Unheated laccase samples in lysis buffer, in which the enzymes were not denatured and remained active, were separated and visualized by CBR staining and zymographic analysis using ABTS substrate. Protein marker (M), native Lcc4 (nLcc4, lane 1), nLcc4 treated with Endo H for 1 h (lane 2), overnight (lane 3), and with an extra supplement of 500 U Endo H in the overnight treatment (lane 4). (C) Optimal pH, optimal temperature and temperature sensitivity determination of nLcc4 and Endo H-deglycosylated Lcc4 (dLcc4). In the temperature sensitivity assay, laccase enzyme was pre-incubated at the indicated temperature for 10 min, and the residual enzymatic activity was then immediately measured. Black rhombi: nLcc4; white rhombi: dLcc4.

To study the effect of the removal of the glycosyl residues from nLcc4, the purified enzyme was de-glycosylated using Endo H to generate Endo H-treated nLcc4 (dLcc4) with only the first GlcNAc moiety left on each *N*-glycosylated Asn residue. nLcc4 protein was digested with Endo H for 1 h or overnight with or without further supplementation of fresh Endo H enzyme. There was no apparent difference in mobility on SDS gel observed for proteins digested with Endo H for 1 h or overnight (Fig 2A, lanes 2–4). Further, the molecular masses of nLcc4 and dLcc4 were determined to be 62 kDa and 56 kDa, respectively, by calibration of their relative mobility distances with molecular weight standards in a 10% SDS-PAGE analysis (Fig 2A). In addition, the activity of nLcc4 and dLcc4 was compared by zymography. As shown in Fig 2B, Endo H (500 U) digestion from 1 hour up to as long as overnight with or without adding another 500 units of Endo H to the reaction mixture resulted in no significant changes in laccase activity in the zymogram.

To confirm the complete reaction of Endo H on nLcc4 protein, dLcc4 was digested by trypsin/Asp-N and subjected to MS analysis. MS results indicated that dLcc4 indeed carried only one GlcNAc on each of the *N*-glycosylation sites Asn^75^, Asn^238^, and Asn^458^ (Table A in S1 File).

### Kinetic analysis of nLcc4 and dLcc4

Next, the kinetic properties of nLcc4 and dLcc4 were determined. When ABTS was used as the substrate, nLcc4 and dLcc4 had the same optimal pH (pH 2.5) and temperature (70°C) (Fig 2C). The heat sensitivity of nLcc4 and dLcc4 was also analyzed and compared. The enzymes were pre-incubated at 25, 30, 40, 50, 60, 70, 80, and 90°C for 10 min and then their residual activities were determined using a standard laccase activity assay at the optimal pH and temperature. Both enzymes exhibited similar heat sensitivity and a dramatic loss of enzymatic activity at temperatures higher than 80°C (Fig 2C).

The detailed catalytic properties of both nLcc4 and dLcc4 were determined with ABTS and 2,6-DMP as substrates at the optimal temperatures and pH values (70°C and pH 2.5, and 60°C and pH 3.5, respectively). The specific activity, turnover rate (*k*_cat_), Michaelis constant (*K*_*m*_), and catalytic efficiency (*k*_cat_/*K*_*m*_) values are shown in Table 3. With ABTS, dLcc4 showed a similar decrease in *k*_cat_ (1.35-fold) and increase in *K*_*m*_ to nLcc4. We therefore concluded that there was no difference in the catalytic efficiency (52 *s* ^**−**1^μM^**−**1^) of the native and de-glycosylated Lcc4. When 2,6-DMP was used as the substrate, only a slightly greater catalytic efficiency (1.18-fold increase) was detected in nLcc4 compared to dLcc4. Overall, the kinetic data indicate that the removal of all of the mannoses leaving only one GlcNAc moiety on Asn^75^, Asn^238^, and Asn^458^ residues in the laccase enzyme did not cause too much deterioration in catalytic activity.

**Table 3:** Kinetic properties of native (nLcc4) and Endo H-deglycosylated lcc4 (dLcc4)^a^.

Protein	Substrate	Specific activity (U/mg)^*b*^	***k***_cat_ (***s*** ^−1^)	***K***_*m*_ (μM)	***k***_cat_/***K***_*m*_ (***s*** ^−1^μM^−1^)	Optimal reaction conditions (°C/pH)
nLcc4	ABTS	2996	3382	65.0±6.5	52	70/2.5
dLcc4	ABTS	2342	2488	48.3±5.5	52	70/2.5
nLcc4	2,6-DMP	119	134	412.1±28.0	0.33	60/3.5
dLcc4	2,6-DMP	114	121	437.1±23.9	0.28	60/3.5

^*a*^ The enzymatic reaction was performed at the respective optimal temperatures and pHs as indicated.

^*b*^ One unit of enzyme activity was defined as the amount of enzyme that oxidizes 1 μmol of substrate per minute under optimal reaction conditions.

### Expression of recombinant wild-type and mutant lcc4 proteins in *P*. *pastoris*

**Fig 3:**
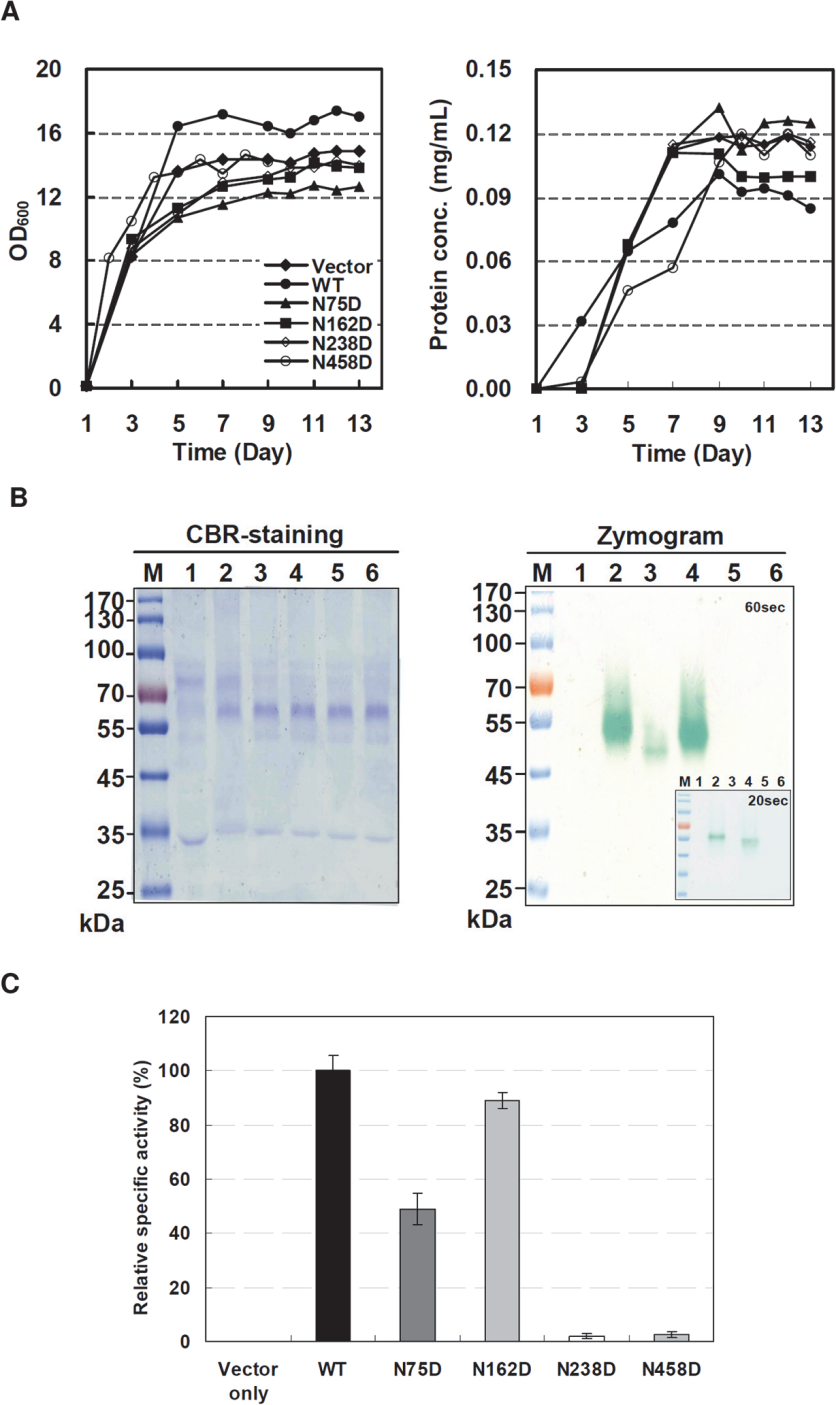
Expression profile of recombinant wild-type and mutant forms of Lcc4 in the cultural media of *P*. *pastoris* X33 cells. (A) Time course of yeast host cell growth and protein expression and concentration. (B) CBR stained gel and zymogram of cultural media. For zymogram analysis, the snapshots of SDS gel were immersed in 2 mM ABTS solution for 20 and 60 s, respectively. Protein marker (M); vector (lane 1); wild-type (lane 2); N75D (lane 3); N162D (lane 4); N238D (lane 5); N458D (lane 6). (C) Relative laccase specific activity in the cultural medium of yeast cells transformed with vector only, wt, N75D, N162D, N238D, and N48D gene construct.

To identify the glycosylation sites that are essential for the laccase protein stability and/or function, yeast *P*. *pastoris* X33 cells were chosen as host for expression of the wild-type (designated rLcc4-WT) and four mutant laccases, N75D, N162D, N238D, and N458D. These recombinant proteins were expressed as extracellular secreted proteins under methanol induction for 13 days. The growth curves and protein expression profiles of the wild-type, four mutant laccases, and the vector plasmid-transformed host cells (vector only) were all similar (Fig 3A). Under equal loading conditions in SDS-PAGE, the protein expression patterns in the cultural media of the wild-type and the mutant enzyme clones were very similar as visualized by coomassie brilliant blue (CBR) staining (Fig 3B). Zymographic analysis showed that the wild-type and N162D laccases revealed their activities within a 20-s substrate ABTS reaction time (Fig 3B *inset*), and the N75D mutant showed activity at around 60 s (Fig 3B). Conversely, the N238D and N458D mutants did not show any detectable band/activity even when the substrate exposure time was extended up to 30 min (data not shown). The specific activity of the crude rLcc4-WT enzyme in the cultural media was further determined as 9 U/mg when ABTS was used as the substrate, and the relative specific activities of the four crude mutant forms of laccase relative to rLcc4-WT (100%) were 49%, 89%, 2%, and 3%, respectively, in N75D, N162D, N238D, and N458D (Fig 3C). These results indicate that mutations of the three confirmed *N*-glycosylation sites N75, N238 and N458 cause changes (> 50% loss) in enzymatic activity, suggesting that glycosylation has an important role in *Lentinus* Lcc4. In particular, maintenance of the first GlcNAc moiety in the enzyme is crucial for the enzyme efficiency.

### Isothermal titration calorimetry determination of nLcc4 kinetic parameters with lignosulfonic acid as the substrate

**Fig 4:**
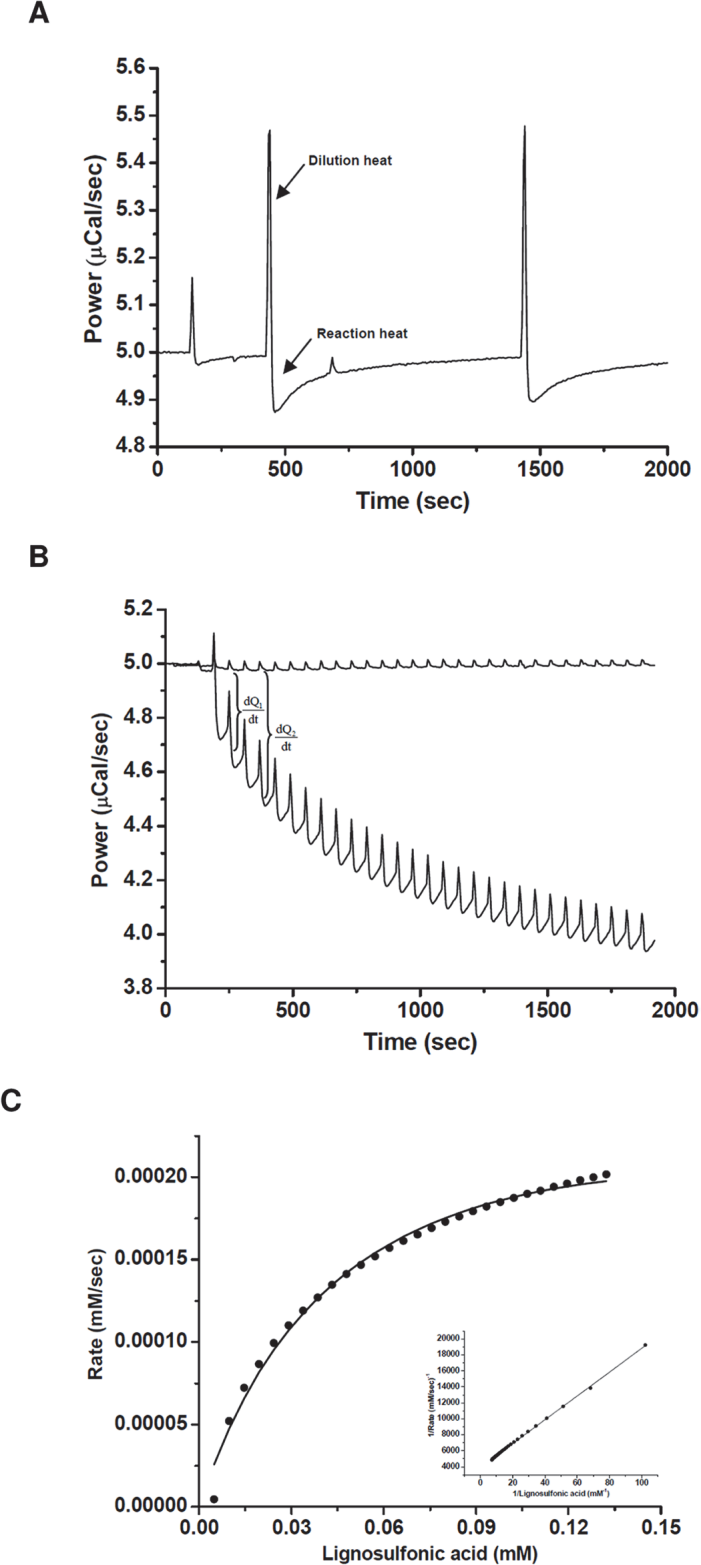
Lignosulfonic acid hydrolysis with *Lentinus* sp. nLcc4 determined by ITC. (A) Thermal power plots as a function of time by single-injection assay, (B) multiple-injection assay, and (C) Michaelis-Menten and Lineweaver-Burk plots of lignosulfonic acid reaction by nLcc4.

We used ITC to determine the catalytic efficiency of nLcc4 on lignosulfonic acid. A sample cell containing 1.39 μM nLcc4 was allowed to reach thermal equilibrium (within 250 s), and 0.1 mM lignosulfonic acid was injected every 1000 s (Fig 4A). The negative deflection indicates that the reaction with substrate lignosulfonic acid (LSA) is exothermic. The apparent enthalpy Δ*H*_app_ of nLcc4 was calculated as -25,040 cal/mol by Eq 1. The multiple-injection method was then applied to determine the catalytic kinetics for lignosulfonic acid. As seen in Fig 4B, increasing the amount of substrate injection into the sample cell resulted in an accompanying increase in thermal power generation by nLcc4, and the dQ/dt value was obtained over the time. The reaction rate (*V*) for the enzymatic reaction was then calculated based on Eq 2. The specific activity, *k*_cat_, *K*_*m*_, and *k*_cat_/*K*_*m*_ of nLcc4 with lignosulfonic acid were determined be 0.207 Umg^**−**1^, 0.234 *s* ^**−**1^, 56.7 ± 3.2 μM, and 0.004 *s* ^**−**1^μM^**−**1^, respectively (Fig 4C).

### Overall structure

To investigate the structural role of glycosylation in nLcc4 protein, the crystal structure of purified nLcc4 was determined at 1.8 Å resolution by X-ray diffraction analysis. The nLcc4 crystal belongs to the orthorhombic space group *P*2_1_2_1_2 and contains two protein molecules (designated as chain A and chain B) per asymmetric unit, similar to observations for crystals of *Lentinus tigrinus* laccase (PDB #2QT6) [6]. The model was refined to a final *R*_work_ of 14.5% and *R*_free_ of 19.7% (Table 2). The cDNA nucleotide sequence and deduced primary amino acid sequence revealed that nLcc4 is composed of 521 amino acids containing a 21-residue signal peptide at the N-terminus that is cleaved during the secretion process. High quality electron density maps allowed us to trace the residues 22 to 521 of the nLcc4 protein chain clearly, and three *N*-glycosylation sites were observed without ambiguity in each protein chain. Furthermore, four copper ions were present in two putative types of copper-binding sites (mono- and tri-nuclear) in the nLcc4 monomer. The structural information for the three *N*-glycosylation sites and four Cu ions binding sites are in good agreement with the deduced primary amino acid sequence of nLcc4 shown in Fig 1B.

**Fig 5:**
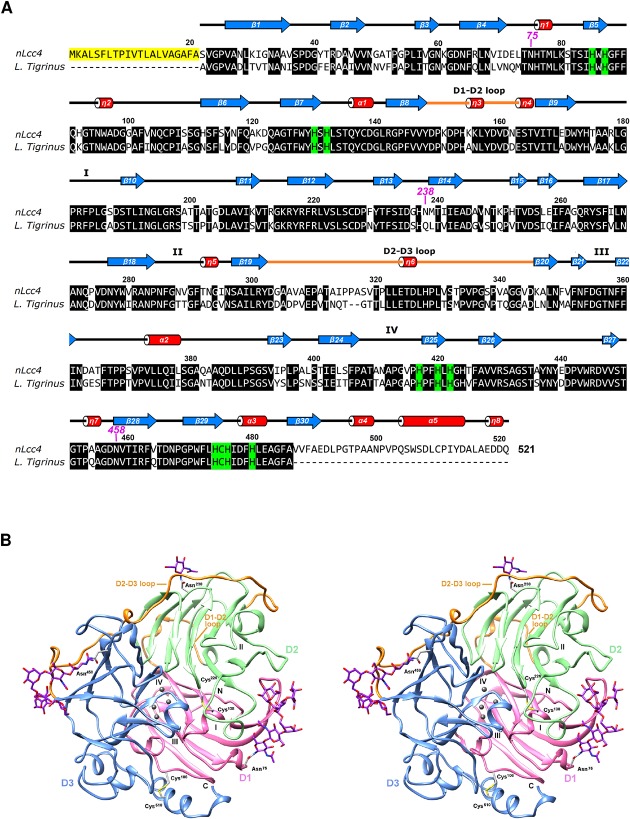
Structure-assisted sequence alignment and stereo views of the structure of nLcc4. (A) Protein sequence alignment of *Lentinus* sp. Lcc4 (PDB #3X1B) and *Lentinus tigrinus* (PDB #2QT6). The signal peptide of *Lentinus* sp. Lcc4 is highlighted in yellow. Residues in α-helices, β -strands, and loops are shown as red cylinders, blue arrows, and lines, respectively. The 3_10_-helices are labeled as η1 to η8. The histidine residues involved in the copper coordination are highlighted in green. The glycosylated Asn residues are marked as magenta numbers. The four substrate binding pocket loops (SBPLs I-IV) and the domain connection loops (D1-D2 and D2-D3 loops) are also indicated. (B) Ribbon diagram of laccase crystal structure, domain 1 (D1), domain 2 (D2) and domain 3 (D3) of laccase are colored pink, green and blue, respectively. Two disulfide bonds (Cys^106^-Cys^510^ and Cys^138^-Cys^226^) and three *N*-glycosylated sites (Asn^75^, Asn^238^, and Asn^458^) are labeled. The licorice representation shows: copper ions in spheres with dark grey and oligosaccharides in sticks with purple carbons. The predicted substrate binding pocket loops (SBPLs I-IV) are also indicated.

**Fig 6:**
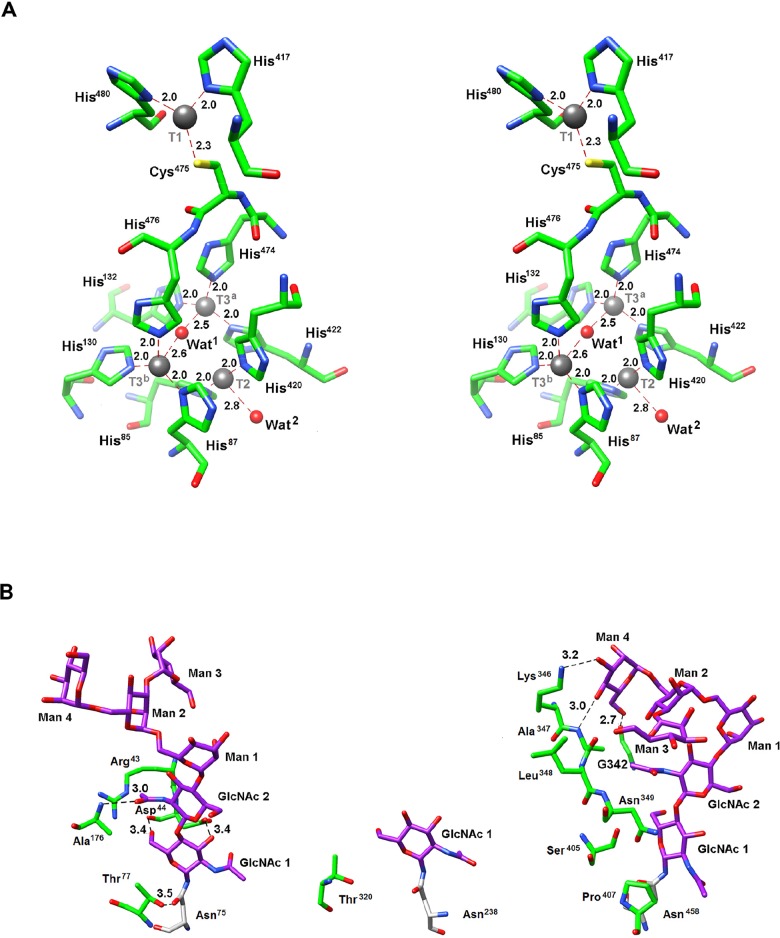
Stereo views of three types of cupper binding sites and glycan moieties of nLcc4. (A) The copper atoms, one type-1 (T1), one type-2 (T2), and two type-3 (T3^a^ and T3^b^), are coordinated to the surrounding histidine, cysteine and the water molecules. Protein residues are shown as a stick model, the oxygen atoms are shown in red, nitrogen in blue, sulfur in yellow, and carbon in green. The four copper ions and water (Wat^1^ and Wat^2^) molecules are represented by gray and red spheres, respectively. (B) The *N*-linked glycans (magenta) on Asn^75^, Asn^238^ and Asn^458^ and the contact residues (green) are shown as stick models. The black dashed lines indicate hydrogen bonds or interactions between *N*-linked glycans and surrounding amino acid residues, in which the amino acid residues 436 to 439 form a β-strand (β 15) structure and are connected to the D2-D3 loop.

The structure of nLcc4 comprises 30 β-strands, 5 α-helices, and 8 3_10_-helices, which fold into three sequentially-arranged cupredoxin-like domains: domain 1 (D1, residues 22–148), domain 2 (D2, residues 163–312) and domain 3 (D3, residues 346–521). Each domain folds into a Greek key *β* -barrel topology (Fig 5A and 5B). Two loops, namely D1-D2 (residues 149–162) and D2-D3 (residues 303–345), link the D1 and D2 domains through β -strands β 8 to β 9, and the D2 and D3 domains through β -strands β 19 to β 20, respectively. Further, D1 is also connected to D2 by a disulfide bridge between Cys^138^ and Cys^226^, while another disulfide bond between Cys^106^ and Cys^510^ not only connects D1 to D3 but also stabilizes the C-terminal helix. nLcc4 also contains one type-1 (T1), one type-2 (T2), and two type-3 (T3^a^ and T3^b^) Cu binding sites. The mononuclear T1 copper is coordinated by His^417^, His^480^, and Cys^475^ and is located approximately 12 Å away from the trinuclear cluster formed by T2 and T3 coppers, which is linked by the electron-transferring Cys-His pathway (Fig 6A). On the other hand, eight histidines are involved in T2/T3 copper cluster coordination, which is embedded in the interface between D1 and D3. The T2 copper is ligated by two histidines (His^85^ and His^420^) and an axial water molecule (Wat^2^), while the two T3 coppers are ligated by three histidines each (His^87^, His^130^, His^476^ and His^132^, His^422^, and His^474^, respectively), and a shared bridging water molecule (Wat^1^). In addition, the mononuclear T1 copper is located at the bottom of the putative ligand binding pocket in D3 and is relatively easily accessible to the solvent, although four substrate binding pocket loops (SBPLs) partially shield it from the solvent (Fig 5B). D1 does not contribute any SBPL to the pocket’s outer surface, but SBPL I (Tyr^173^ to Asp^188^) on D2 actually acts as a link between the binding pocket surface and D1. SBPL II (Asn^285^ to Asn^296^) is also part of D2, while SBPL III (Asp^355^ to Asn^358^) and SBPL IV (Ala^408^ to His^417^) are parts of D3. SBPL III connects β 21 to β 22, following the D2-D3 loop and an extension of β 20. Further, SBPL III also protrudes outwards, towards D1. SBPL IV, on the other hand, is located between β 24 and β 25, and is relevant both to the surface of the putative binding pocket and to copper coordination via His^417^ (Fig 5A and 5B).

Although four *N*-glycosylation sites, Asn^75^, Asn^162^, Asn^238^, and Asn^458^ were predicted based on the consensus sequence Asn-X-Ser/Thr in the deduced nLcc4 protein sequence (Fig 1B), the density maps clearly indicated that only Asn^75^ in D1, Asn^238^ in D2, and Asn^458^ in D3 are *N*-glycosylated, which is consistent with the MS data. Two polypeptide chains A and B were observed in the crystal structure. Chain A contains two *N*-acetyl-D-glucosamines (GlcNAc 1 and GlcNAc 2) and four branched α-D-mannose moieties (Man 1, Man 2, Man 3, and Man 4) (N2M4) on both the Asn^75^ and Asn^458^ residues. In chain B, Asn^75^ contains N2M4 (GlcNAc 1, GlcNAc 2, Man 1, Man 2, Man 3, and Man 5), but Asn^458^ contains N2M5 (GlcNAc 1, GlcNAc 2, Man 1, Man 2, Man 3, Man 4, and Man 5). In both chain A and B crystal structures, we can only observe one GlcNAc bound to Asn^238^ (data not shown). The N2M4 oligosaccharides on Asn^75^ in chain A interacted with the protein mainly via the two basal GlcNAc moieties (Fig 6B). For GlcNAc 1 on Asn^75^, while OH6 was hydrogen-bonded to the peptide carbonyl of Asp^44^, OH3 interacted with the carboxylic group of the same amino acid. Further, Thr^77^ interacted via a hydrogen bond with the amide group in Asn^75^, and via a carbon-carbon stacking with C1 atom of GlcNAc 1 on Asn^75^. GlcNAc 2 on Asn^75^ interacted with Arg^43^ via a single hydrophobic contact between the aliphatic portion of the residue side-chain and C3 atom of GlcNAc 2. The only other interaction between GlcNAc 2 on Asn^75^, and the protein was a hydrogen bond between amide oxygen of GlcNAc 2 and the peptide proton of Ala^176^. None of the Man moieties in glycan 75 appeared to interact with any protein element. In addition, the only observable GlcNAc on Asn^238^ was stabilized by a single hydrophobic contact between C6 atom of GlcNAc and Thr^320^ Cγ atom. The glycan bound to Asn^458^ did not show any strong interaction between the protein and the two GlcNAc moieties. The methyl group of GlcNAc 1 on Asn^458^ interacted with the aliphatic side-chain of Pro^407^, while the C5 atom of GlcNAc 1 was stacked against the Cβ atom of Ser^405^. GlcNAc 2 on Asn^458^ had a long range hydrophobic interaction between its amide carbon and the Cβ atom in Asn^349^; and the methyl group of GlcNAc 2 on Asn^458^ had hydrophobic contacts with the Cα atom of Leu^348^ and with the Cα and Cβ atoms of Ala^347^. On the other hand, the terminal Man 4 moiety on Asn^458^ in chain A was anchored firmly to the protein, resulting in a charged-hydroxyl interaction between OH3 and the side-chain of Lys^346^, which directly links the N2M4 N-glycan on Asn^458^ to the D2-D3 loop. Two hydrogen bonds further stabilized Man 4 on Asn^458^, one between OH4 of Man 4 and the amide proton of Ala^347^, and the other between OH6 of Man 4 and the carbonyl oxygen of Gly^342^. All the interactions between the glycosyl moieties and specific amino acids in nLcc4 (chain A) are depicted in Fig 6B.

### Molecular dynamics simulations of nLcc4

Kinetically, we observed that when one GlcNAc was left in each of the three authentic *N*-glycosylated Asn residues, most of the enzymatic activity was retained; conversely, complete removal of the whole sugar moiety at either the 238 or 458 *N*-glycosylation sites caused dramatic loss of laccase activity. We thus conducted a study of the structural basis of glycan-dependent laccase activity by molecular dynamics (MD) simulations of different glycosylation states. The conditions chosen for all simulations mimicked the experimental parameters and optimal conditions (pH 3.0 and 30°C) for the degradation of lignosulfonic acid.

**Fig 7:**
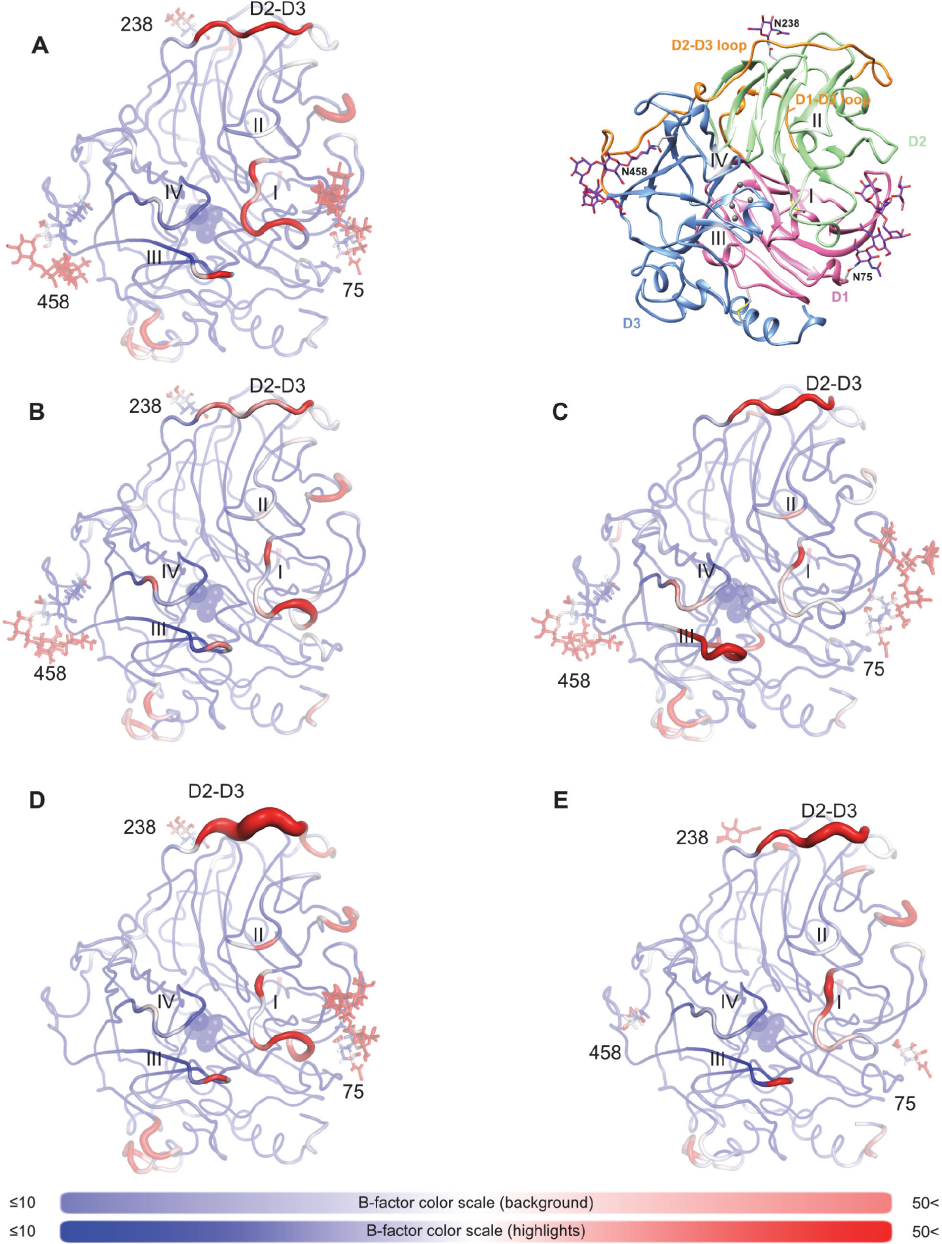
Molecular dynamics simulations of Lcc4 in different glycosylation states at pH 3.0 and 303 K. All structures represent average structures along an 18 ns production MD simulation. Molecules are colored by B-factor, as calculated for the entire simulation. Blue regions indicate low B-factors, and therefore, low molecular motion, while red regions are flexible, and have high B-factors. Deep blue indicates B-factors of 10 Å^2^ or lower, while bright red indicates values equal to, or higher than, 50 Å^2^. The thickness of the represented structure is also proportional to the B-factor value. (A) Simulation of nLcc4 on the left (Video A in S2 File). On the right, the general structure of nLcc4, highlighting the original crystal structure. (B) Partially de-glycosylated laccase without the Asn^75^ N-glycan (Video B in S2 File). (C) Partially de-glycosylated laccase without the Asn^238^ N-glycan (Video C in S2 File). (D) Partially de-glycosylated laccase without the Asn^458^ N-glycan (Video D in S2 File). (E) dLcc4, containing only the first GlcNAc at each of the confirmed *N*-glycosylated sites (Video E in S2 File).

**Fig 8:**
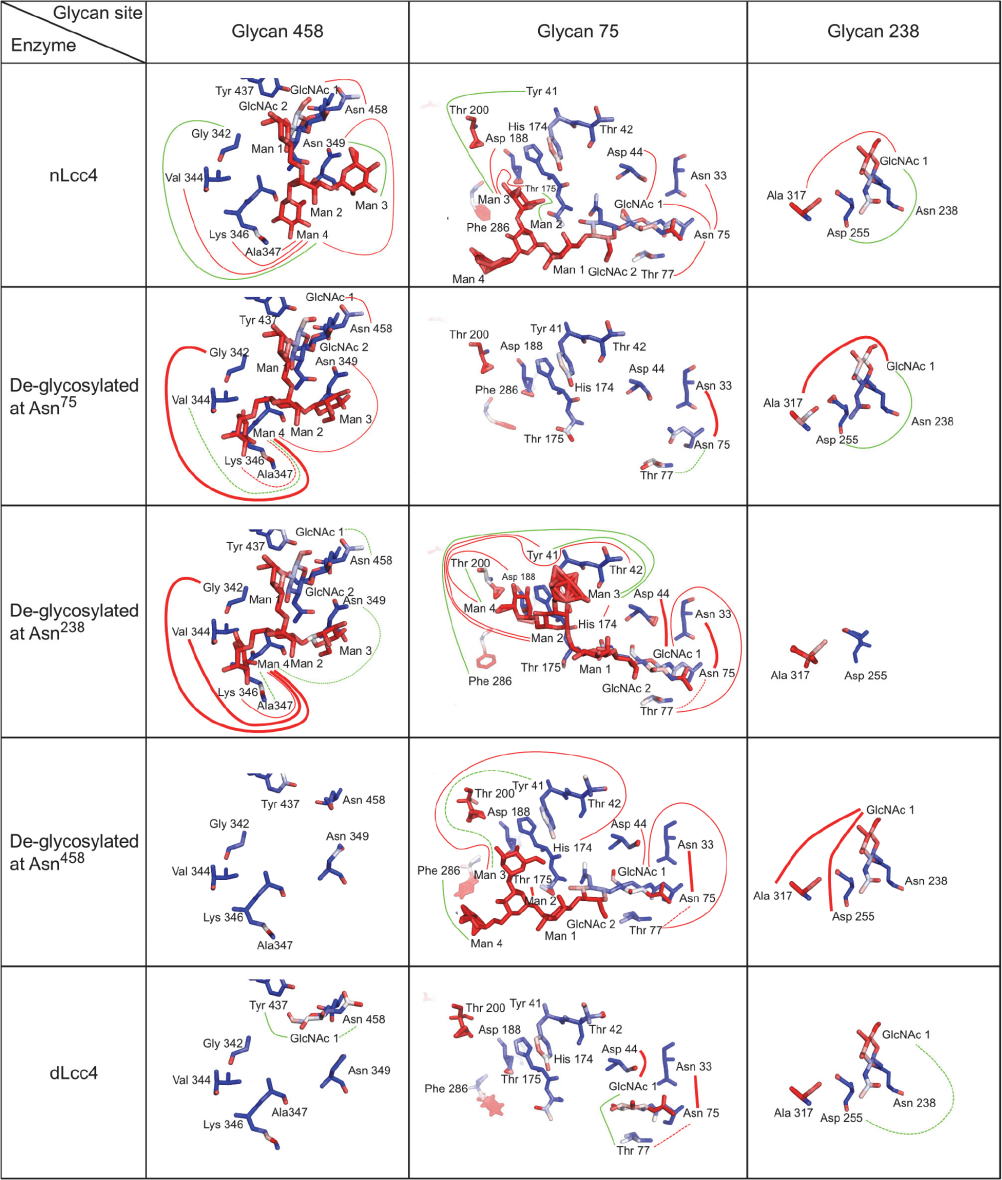
Hydrogen bond network of the three glycans under different glycosylation conditions. Hydrogen bonds are depicted as lines stretching from one partner to the other. Red lines represent strong bonds (occupancy >10% of the total simulation time) and green lines represent weak bonds (occupancy <10% of the total simulation time). Further, de-glycosylation simulation results are compared to the native state by the representation of weaker hydrogen bonds as dotted lines, and stronger hydrogen bonds with thicker lines. All data evaluation was performed with ptraj.

In the native state simulations, each sub-domain motif (D1-D3) within the laccase structure had one glycan moiety attached as shown in the crystal structure (Fig 5B). In Fig 7A, average models of nLcc4 derived from the MD simulations are color-coded by MD-derived per-residue B-factors. As depicted in Fig 7A and Video A in S2 File, the glycans at Asn^238^ and Asn^458^ interacted more tightly with the protein through several strong hydrogen bonds (Fig 8), which is in agreement with the crystal structure (Fig 6B). The mannose moieties of glycan 75 were found to have an extended and flexible conformation during the first few nanoseconds of the simulation; in addition, glycan 75 interacted transiently with SBPL I. In contrast, glycan 458 was observed to interact extensively with SBPL III during the entire simulation which forms the rigid conformation of glycan 458 and results in steric hindrance on SBPL IV and (Fig 7A and Video A in S2 File). Glycan 238 did not contact any of the SBPLs directly, but linked the D2-D3 loop to D2 via the Asp^255^ side-chain and the peptide bonds of Ala^317^ in the D2-D3 loop (Fig 8).

The contributions of each glycan to the overall stability of the laccase were further studied by performing a set of three simulations in which only two of the three glycosylation sites were occupied (Fig 7B–7D, and Videos B-D in S2 File). When glycan 75 was removed, no large long-range effects could be observed, whereas SBPL I was only slightly destabilized compared with the fully glycosylated form simulation, suggesting that the removal of glycan 75 has a more local effect reflecting the partial loss of enzymatic activity in the kinetic analysis (Fig 7A and 7B and Video B in S2 File). In addition, no large changes in the hydrogen network of other glycans occurred after the removal of glycan 75 (Fig 8). On the other hand, when glycan 238 was removed, a large number of long-range effects are transmitted via the destabilization of the D2-D3 loop, resulting in the destabilization of SBPLs II, III, and IV (Figs 7C and 8 and Video C in S2 File). Furthermore, a profound reconfiguration of the hydrogen bonding network of glycan 75 was revealed. The removal of the glycan on position 458 closely mirrored the removal of the one on position 238, with SBPLs I, II, and IV exhibiting increased motion (Fig 7D and Video D in S2 File). The D2-D3 loop also underwent a strong conformational change, as the interaction between GlcNAc on position 238 and the carbonyl backbone of Ala^317^ in the D2-D3 loop became stronger (Fig 8), and the entire loop shifted away from the vicinity of the binding pocket (Video D in S2 File). When glycan 458 was removed, it further mimicked de-glycosylation at position 238, as a shift of the D2-D3 loop was also observed to cause the mannose moieties of glycan 75 to adopt a new conformation (Fig 8). These molecular dynamics simulation observations mirrored the results seen with the recombinant laccase proteins, i.e., that the complete removal of glycans at Asn^238^ and Asn^458^ by site-directed mutagenesis caused a dramatic loss (> 95%) of enzymatic activity, and approximately of 50% enzymatic activity was retained in N75D mutant compared to wild-type enzyme.

Because partial de-glycosylation did not have a significant effect on Lcc4 activity, we also simulated a partially de-glycosylated laccase, in which only the first GlcNAc moiety of each site remained (Fig 7E and Video E in S2 File). Interestingly, the GlcNAc residues appeared to be sufficient for the stabilization of the SBPLs III and IV, with the protein being almost as stable as the fully glycosylated protein. Further, SBPL I retracted into a position which was similar to the one adopted in the absence of glycan 75, further suggesting that the presence of glycans on Asn^75^ is not as critical as on the other two glycosylated sites. Since position 238 was originally only occupied by a single GlcNAc moiety, partial de-glycosylation on other two sites did not affect the local structure of this site; however, the absence of most of the sugar moieties on Asn^75^ and Asn^458^ did affect their local hydrogen bonding networks. The single GlcNAc left on Asn^458^ resulted in a new weak hydrogen bond with Tyr^437^ (Fig 8), which in turn interacted via π -π stacking with Tyr^437^. As Tyr^437^ is in direct contact with loop D2-D3, the modified hydrogen bond network of the partially de-glycosylated site 458 might have the same stabilization effects as the full glycan, albeit via a different pathway.

## Discussion

In this study, we identified a novel isoform of laccase (nLcc4) from a Formosan Basidiomycete white-rot fungus *Lentinus* sp., and its encoded gene and crystal structure were characterized. In comparison with the primary amino acid sequences of seven other Basidiomycete fungal laccases, four highly conserved Cu ion binding regions and two highly conserved *N*-glycosylation sites Asn^75^ and Asn^458^ were observed in the Asn-X-Ser/Thr consensus sequence (Fig 1B). Although glycosylation is quite often observed in laccase proteins of fungal origins, the exact role of glycosylation in these enzymes is not yet full-understood. In an attempt to elucidate the structural and functional roles of glycosylated amino acid residues and glycol moieties in the enzyme, we first employed LC-MS/MS to confirm the glycosylation sites in nLcc4. Using deamidated residues of Asn (Asp) with one ^18^O substitution of the predicted *N*-glycosylation sites, and MS analysis of PNGase F/Asp-N deglycosylated tryptic peptides, we were able to confirm that Asn^75^, Asn^238^ and Asn^458^ are the three glycosylated residues in nLcc4. These glycosylated residues were further confirmed by the three-dimensional structure of the enzyme solved by X-ray diffraction. The complementary glycan structure (Man_5_GlcNAc_2_, or N2M5) by integration of chain A and chain B structures in Asn^75^ and Asn^458^ are very typical of Basidiomycetes laccases [36], and only one GlcNAc was observed attached to Asn^238^ (Fig 6B).

Interestingly, Endo H-digested nLcc4 (dLcc4) which had only one GlcNAc remaining attached to each of the three glycosylated residues of the enzyme caused little or no deterioration in enzymatic activity. Vite-Vallejo and coworkers (2009) suggested that Endo F1-deglycosylation leaving one GlcNAc in *P*. *sanguineus* laccase could result in the enzyme being less stable at temperatures below 50°C, and decrease the activity at 20°C [12]. These phenomena were not observed in *Lentinus* Lcc4. However, one single mutation (N75D, N238D or N458D) to remove the whole glycan on one of the three glycosylated residues could cause significant loss of activity compared to the recombinant wild-type enzyme (rLcc4-WT). In order to assess the structural integrity of the N to D mutations at Asn75, Asn238, or Asn458 we further ran an MD simulation to confirm whether the N to D mutation at Asn75, Asn238, and Asn458 would cause any effect on in its local structural environment. MD simulation on the protein model with either Asp or Asn at residues 75, 238 and 458, respectively, without the concern of the presence of glycosyl groups, were carried out. In Figure A in S1 File, it is easy to see that the switch from N to D in the N238 and N458 sites does not perturb the residue's micro-environment. On the other hand, at site N75, we detected very minor differences in the microenvironment upon N to D mutation. These differences, however, do not seem to be extensive, and did not change the overall behavior of the simulation. In addition, we calculated the percentage of protonation at each mutation site by H++ server. In the acidic reaction conditions (pH 2.5), Asp75, Asp238, and Asp458 show an 86%, 96%, and 99% protonation fraction (Table C in S1 File). Based on these data, we can assume these aspartates were protonated during the enzyme reaction, suggesting that the replacement of Asn by Asp did not affect the structural integrity or protein property of the enzyme. Therefore, we can conclude that the significant decreases in enzyme activity in the N75D, N238D and N458D mutants are mainly the result of the removal of the sugar moiety, and not due to charge derived alterations in the protein structure. In parallel, we also performed a simulation where N162 had been substituted by aspartate, although this is not a glycosylation site in nLcc4. When the wild-type average structure (colored in yellow) is compared with the mutant one (colored by B-factor), it appears that N-terminal region, which is in close proximity to N162, was stabilized by the mutant in its deprotonated state (~20% fraction at pH 2.5 based on H++ server calculation). As the N-terminus is an integral part of domain D1, perhaps the lack of flexibility in this domain might account for the slightly lower activity.

On the other hand, our kinetic results suggest that the degree of glycosylation in protein plays a critical but different role in the catalytic function or mechanism within fungal laccases. Of note, we observed that the protein yield and laccase activity (~600 U/L) of recombinant Lcc4 expressed and detected in the culture medium of the *P*. *pastoris* host cell system were considerably low compared to the native laccases (~58,000 U/L) expressed in the culture medium of its *Lentinus* origin. This phenomenon although unfortunate, is unsurprising as it is commonly observed in heterogeneous expression of other fungal laccases in *P*. *pastoris* [37]. We suggest that, in our laccase, it might be partly due to the hyperglycosylation of the enzyme in yeast cells, as the molecular mass was increased in rLcc4 (64 kDa) compared to nLcc4 (62 kDa) as determined by SDS-PAGE (data not shown). According to the suggested length of oligosaccharide chains commonly added to foreign proteins expressed in the *Pichia* host cell system [38], we conducted molecular dynamics simulations of rLcc4 protein and found that lengthening the high-mannose oligosaccharides (GlcNAc2Man14) generally resulted in lower B-factors for the entire protein (Figure B in S1 File), especially in the copper bonding sites and the four SBPLs themselves, suggesting the increased rigidity in protein structure. This might result in deterioration in the flexibility of conformational changes for substrate binding during the catalytic reaction cycles of the enzyme. Together, the MD simulation result implies that the substrate binding pocket architecture does not benefit from the increased protein stability brought by hyperglycosylation, and instead, some stereo-hindrance might occur that leads to improper protein folding or substrate binding.

To investigate the structural role of glycosylation in nLcc4 in a more detailed manner, molecular dynamics simulations were carried out on nLcc4 structures with different degrees of de-glycosylation. Overall, in this work our MD simulations correlated nicely with the kinetic data, and provide a structure-based explanation for the observed glycan-dependent activity which involves increased molecular motion in three of four key loops surrounding the active site of the enzyme. In comparison to previous MD-based laccase thermostability studies, which suggested glycan-protein interactions had important local effects [13]; in this study, we use a different approach for the glycan and copper parameters. The carbohydrate specialized GLYCAM06 force field is extremely robust, and even though non-scaled 1–4 interaction energies might be a weakness in the simulation of free glycans, it has been shown that these have a very minor effect on protein-attached carbohydrates [39]. For the copper parameters, we chose to use a bonded plus electrostatics model, which has the advantage of taking into account the charge transfer between the metals and interacting amino-acids in non-polarizable force fields [30]. The main drawback of this model is that it locks the protein into a single redox state for each of the coppers, and therefore will preclude the simulation of complex conformational changes derived from the catalytic cycle. However based on these choices, in this study we are able to address and relate glycosylation to global and local changes within the protein architecture, taking into account the tight metal-amino acid relationship in and around the active site.

**Fig 9:**
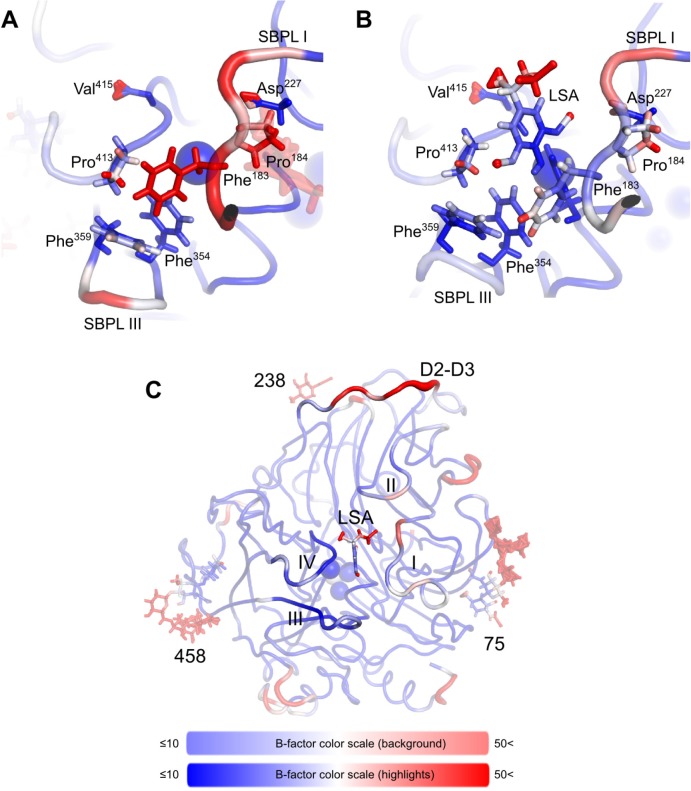
Molecular dynamics simulations of nLcc4-lignosulfonic acid complex. (A) Detail of the free form of nLcc4 binding pocket, corresponding to the average structure in Fig 7A. (B) Detail of the average simulated nLcc4 binding pocket bound to lignosulfonic acid (LSA), highlighting the stabilized Phe^183^ and Asp^227^ and the relative conformational changes of SBPLs I and III. (C) Full average structure of the computed nLcc4-lignosulfonic acid complex simulation. Structures represent average structures along an 18 ns production MD simulation. Molecules are colored by B-factor, as calculated for the entire simulation with the same palette as in Fig 7. The thickness of the represented structure is also proportional to the B-factor value.

On SBPL I, simulated B-factors indicated that molecular motion was increased by the presence of glycan 75 (Fig 7), which experimental kinetic studies indicated is far less critical for enzyme activity than either glycan 458 or glycan 238. These two glycans interact directly with the D2-D3 loop, a lengthy and important loop composed of 43 amino acid residues (8% of overall protein primary sequence), which crosses over two structural domains of the laccase, and also connects two SBPLs (II and III) in the enzyme (Fig 7). When either glycan 238 or 458 is removed, the molecular motion of D2-D3 loop is increased, resulting in extensive changes being transmitted along the entire protein. In the absence of glycan 458, the interaction between glycan 238 and the D2-D3 loop is strengthened (Figs 7D and 8 and Video D in S2 File), with the latter tending to recede from its corresponding position in the fully glycosylated form. Conversely, when glycan 238 is removed, SBPLs III and IV, which are in the vicinity of glycan 458, are strongly affected. Furthermore, the hydrogen bonding network of 458 is profoundly changed with the removal of glycan 238, as a result of a conformational change in the former (Fig 8). The D2-D3 loop is connected to SBPL III via β-strands β20 and β21, which are part of the same β-sheet as the β-strands connected by SBPL IV (Fig 5A). Thus, the conformational changes in the D2-D3 loop affect the binding pocket surface, suggesting that the D2-D3 loop underlies the mechanism by which glycans 238 and 458 play significant roles in maintaining enzymatic activity. While each of these glycans affects the SBPLs in their vicinity or the molecular motions on the D2-D3 loop, we propose that, when one of them is completely removed, it is the disruption of their combined interaction with the D2-D3 loop that has the biggest impact on the protein activity. Whereas glycosylation site 458 is highly conserved across all related laccases, site 238 is not. A close inspection of the structures of all related laccases (Table B in S1 File) which do not present glycan 238 reveals alternative strategies to stabilize the D2-D3 loop. For example, the laccases of *B*. *aclada* (PDB #3SQR), *P*. *cinnabarinus* (PDB #2XYB), and *M*. *albomyces* (PDB #2Q9O) have nearby, alternative glycosylation sites which also interact with the D2-D3 loop. In others, the position equivalent to Asn^238^ presents amino acids, which yield long range interactions with the D2-D3 loop; this is the case for the laccase from *S*. *ochraceum*, in which the glycosylation site has been substituted by an aspartate, which in turn interacts with the D2-D3 loop via Lys^302^. Finally, the region of the D2-D3 loop which is in the direct proximity of glycan 238 in nLcc4 is unusually hydrophobic (72% of the amino acids at positions 302 to 323 are either aliphatic, or aromatic). For laccases where the position 238 is not glycosylated, and indeed in some of the laccases with alternative glycosylation as well, this region is much more hydrophilic (52% aliphatic amino acids on average, 50% aliphatic amino acids for those laccases without any glycosylation whatsoever). As a result, we would like to suggest that the higher capacity for hydrogen bonding between the D2-D3 loop and the underlying protein surface in laccases without glycan 238 compensates for a less tightly coordinated hydration sphere. To further support this proposed mechanism of action of laccase, we ran simulations of lignosulfonic acid (LSA) docked into the binding pocket of nLcc4 (Fig 9 and Video F in S2 File). These were performed using the same parameters for the copper atoms as developed for the empty pocket, and therefore did not take any metal-ligand interactions into account. When compared to the empty binding pocket, when LSA is bound, SBPL I becomes more flexible, while SBPL III becomes more rigid. While Phe^183^ is involved in a π-π interaction with the aromatic wing of LSA, the catalytic residue Asp^227^ is also stabilized by the substrate, suggesting that glycosylation might contribute to substrate binding affinity by pre-priming the four SBPLs (Fig 9C).

**Fig 10:**
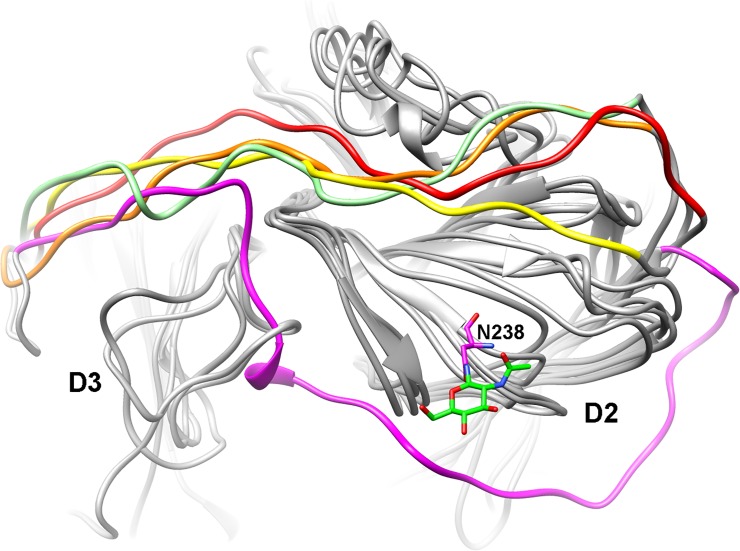
Comparisons of D2-D3 loop orientations. Four bacterial laccases (PDB #2FQG, #1GSK, #2YAE and #4F7K) are superimposed with nLcc4. The D2-D3 loop in nLcc4 is threaded in direct proximity to the binding pocket; the D2-D3 loops in the other four bacterial laccases show the same orientation to connect D2 and D3 domains and pass near the N-terminus, where they cannot interact with any of the SBPLs. The D2-D3 loops from 2FQG, 1GSK, 2YAE, 4F7K and nLcc4 are colored orange, green, yellow, red and magenta, respectively.

Furthermore, we compared and superimposed the overall structure of our *Lentinus* sp. nLcc4 with the only other known crystal structure of a *Lentinus* laccase (from *L*. *tigrinus*, PDB #2QT6) [6]. The primary amino acid sequences of both *Lentinus* laccases revealed 75.7% identity with 465 amino acid residues (comparing from residue 22 to residue 486 of nLcc4), and had a similar composition of three structural domains (D1 to D3), two domain joining loops (D1-D2 and D2-D3), and four substrate binding pocket loops (Fig 5A). When superimposed, the overall structures of both proteins are similar with an r.m.s.d. value of 0.41 Å for 488 C^α^ atoms. Although both enzymes have different glycosylation sites, i.e., Asn^75^, Asn^238^ and Asn^458^ in nLcc4, and Asn^75^, Asn^397^ and Asn^458^ in *L*. *tigrinus* laccase, the D2-D3 loop in both enzymes is orientated in the same direction (data not shown). We further compared nLcc4 with the structures of 13 other non-redundant Basidiomycete fungal laccases deposited in the RCSB Protein Data Bank (http://www.rcsb.org/pdb/home/home.do), which are all glycosylated proteins and with 56–78% identity, we observed that the D2-D3 loop among them are all orientated in the same direction. We further compared the nLcc4 structure with the crystal structure of non-glycosylated laccases from four bacteria (PDB #2FQG, #1GSK, #2YAE and #4F7K) [40,41]. It is interesting to note that the corresponding D2-D3 loops in these four bacterial laccases are located at the opposite side of the binding pocket in the protein structure compared to nLcc4 (Fig 10) and other 13 glycosylated Basidiomycete fungal laccases (data not shown). Our study results and the structural comparison within fungal laccase proteins demonstrate that glycan moieties contribute significantly to maintenance of the D2-D3 orientation in fungal laccases, an orientation that is not observed in bacterial laccase lacking in glycosylation. From these observations we conclude that the orientation of D2-D3 loop and its interactions with glycan moieties play a crucial role in mediating glycosyl-fungal laccase activity. We therefore propose that a loop-glycan interaction mechanism supports fungal laccase activity. Mutagenesis and the functional role of the specific amino acid residues involved in the loop-glycan interaction as observed from the crystal structure and MD analysis of nLcc4 warrant further investigation to shed more light on fungal laccase catalysis.

## Accession Numbers

The coordinates and structure factors for nLcc4 were deposited with the Protein Data Bank under accession number **3X1B**.

The full-length *lcc4* gene was deposited in GenBank with accession number **KF836751**.

## Supporting Information

S1 File Table A in S1 File. Trypsin-digested and PNGase F/Endo H-deglycosylated peptides. **Table B in S1 File**. Comparison between the D2-D3 loop-glycan 238 regions of homologous laccases of known structure. **Table C in S1 File**. Calculated percentage of protonation at the corresponding pH. **Figure A in S1 File**. **Asn to Asp mutant MD simulation**. The micro-environments surrounding each of the glycosylation sites are colored as follows: aspartate (Asp) mutants are colored by B-factor, de-glycosylated wild-type is colored grey, and wild-type fully glycosylated nLcc4 is colored yellow. A. N/D 458, B. N/D 238, C. N/D 75, and D. N/D 162. The MD simulation parameters are set as shown in Fig 7 but without the background adjustment. **Figure B in S1 File. Simulation model of hypothetical glycosylated rLcc4 protein expressed in the Pichia host cell system**. In Pichia, it has been suggested that proteins are hyperglycosylated with mannose-rich N-glycans (on average with Mannoses 9–14) [2]. Left side: hyperglycosylated rLcc4 modeled with 14 mannoses (Man 14), colored by simulated B-factor. Right side: Simulated B-factor values for the copper ions and SBPLs for rLcc4 under native (GlcNAc2Man5) and *Pichia* glycosylation (GlcNAc2Man14). *Pichia* glycosylation consistently reduces plasticity around the binding pocket.
(DOC)

S2 File Video A in S2 File. **Simulation of nLcc4. Video B in S2 File**. Simulation of partially de-glycosylated laccase without the Asn75 N-glycan. **Video C in S2 File**. Simulation of partially de-glycosylated laccase without the Asn238 N-glycan. **Video D in S2 File**. Simulation of partially de-glycosylated laccase without the Asn458 N-glycan. **Video E in S2 File**. Simulation of dLcc4, containing only the first GlcNAc at each of the confirmed *N*-glycosylated sites. **Video F in S2 File**. Simulations of lignosulfonic acid (LSA) docked into the binding pocket of nLcc4.
(RAR)
