# Supplementary material for: Structural and Functional Roles of Glycosylation in Fungal Laccase from Lentinus sp
Source: PLoS One. 2015 Apr 7;10(4):e0120601. doi: 10.1371/journal.pone.0120601 (PMC4388643; doi:10.1371/journal.pone.0120601)
Supplement: S1 File — Trypsin-digested and PNGase F/Endo H-deglycosylated peptides. Table B in S1 File. Comparison between the D2-D3 loop-glycan 238 regions of homologous laccases of known structure. Table C in S1 File. Calculated percentage of protonation at the corresponding pH. Figure A in S1 File. Asn to Asp mutant MD simulation. The micro-environments surrounding each of the glycosylation sites are colored as follows: aspartate (Asp) mutants are colored by B-factor, de-glycosylated wild-type is colored grey, and wild-type fully glycosylated nLcc4 is colored yellow. A. N/D 458, B. N/D 238, C. N/D 75, and D. N/D 162. The MD simulation parameters are set as shown in Fig 7 but without the background adjustment. Figure B in S1 File. Simulation model of hypothetical glycosylated rLcc4 protein expressed in the Pichia host cell system. In Pichia, it has been suggested that proteins are hyperglycosylated with mannose-rich N-glycans (on average with Mannoses 9–14) [2]. Left side: hyperglycosylated rLcc4 modeled with 14 mannoses (Man 14), colored by simulated B-factor. Right side: Simulated B-factor values for the copper ions and SBPLs for rLcc4 under native (GlcNAc2Man5) and Pichia glycosylation (GlcNAc2Man14). Pichia glycosylation consistently reduces plasticity around the binding pocket. (DOC) [file pone.0120601.s001.doc]

Supporting Information for

**Structural and Functional Roles of Glycosylation in Fungal Laccase from *Lentinus* sp.**

**Manuel Maestre-Reyna^1^**^¶^**, Wei-Chun Liu^2^**^¶^**,** **Wen-Yih Jeng^3,4^, Cheng-Chung Lee^1,4^, Chih-An Hsu^1^, Tuan-Nan Wen^5^, Andrew H.-J. Wang ^1,3,4,6^* and Lie-Fen Shyur^2,7^***

^1^ Institute of Biological Chemistry, Academia Sinica, Taipei, Taiwan

^2^ Agricultural Biotechnology Research Center, Academia Sinica, Taipei, Taiwan

**^3^** Center for Bioscience and Biotechnology, National Cheng Kung University, Tainan, Taiwan

**^4^** Core Facilities for Protein Structural Analysis, Academia Sinica, Taipei, Taiwan

**^5^** Institute of Plant and Microbial Biology, Academia Sinica, Taipei, Taiwan

**^6^** Ph.D. Program for Translational Medicine, College of Medical Science and Technology, Taipei Medical University, Taipei, Taiwan

**^7^** Ph.D. Program for Translational Medicine, Kaohsiung Medical University, Kaohsiung, Taiwan

^¶^: These authors contributed equally to this work

*: Co-Corresponding authors

[ahjwang@gate.sinica.edu.tw](mailto:ahjwang@gate.sinica.edu.tw) (AHJW); [lfshyur@ccvax.sinica.edu.tw](mailto:lfshyur@ccvax.sinica.edu.tw) (LFS)

**The total number of pages is 12 in the “Supporting Information”, which includes Table A and spectral data from MS analysis, Tables B-C, Figures A-B, and References.**

**Table A**

| **Trypsin-digested and PNGase F-deglycosylated peptides** | |  | |  | |  |  |  |  |
| --- | --- | --- | --- | --- | --- | --- | --- | --- | --- |
| **Sample Name** | | **nLcc4** | | | |  | | | |
|  | **Sequence** | **Charge** | **Monoisotopic m/z** | | **Ion Score/XCorr** |  | | | |
| **Peptide A (N->D)** | LNVIDELTnHTMLK | +3 | 548.62354 Da (+0.06ppm) | | 68/2.95 |  | | | |
| **Peptide A (N->D, Moxi)** | LNVIDELTnHTmLK | +3 | 553.95471 Da  (-0.77 ppm) | | 49/2.85 |  | | | |
| **Peptide C’ (N->D)** | DVVSTGTPAAGDnVTIR | +2 | 838.42139 Da  (-1.73 ppm) | | 92/4.09 |  | | | |
| **Asp-N digested and PNGase F-deglycosylated peptides** | | | | | |  | | | |
|  | **Sequence** | **Charge** | **Monoisotopic m/z** | | **Ion Score/XCorr** |  | | | |
| **Peptide B (N->D, Moxi)** | DGHnmTIIEA | +2 | 560.24640 Da  (-1.60 ppm) | | n.a./2.17 |  | | | |
| **Peptide C (N->D)** | DnVTIRFVT | +2 | 534.28400 Da  (-1.01 ppm) | | 41/2.72 |  | | | |
|  |  |  |  | |  |  | | | |
| **Trypsin-digested and Endo H-deglycosylated peptides** | | | | | | | | | |
| **Sample Name** | | **dLcc4** | | | |  | | | |
|  | **Sequence** | **Charge** | **Monoisotopic m/z** | | **Ion Score/XCorr** |  | | | |
| **Peptide A-HexNAc** | LNVIDELTnHTMLK | +2 | 922.47736 Da  (+0.20 ppm) | | 60/3.94 |  | | | |
| **Peptide A-HexNAc (Moxi)** | LNVIDELTnHTmLK | +2 | 930.47406 Da  (-0.61 ppm) | | 63/3.20 |  | | | |
| **Peptide C′-HexNAc** | DVVSTGTPAAGDnVTIR | +2 | 938.46741 Da  (-1.05 ppm) | | 95/3.92 |  | | | |
| **Asp N-digested and Endo H-deglycosylated peptides** | | | | | |  | | | |
|  | **Sequence** | **Charge** | **Monoisotopic m/z** | | **Ion Score/XCorr** |  | | | |
| **Peptide B′-HexNAc** | DPFYTFSIDGHnMTIIEA | +2 | 1137.51685 Da  (-0.33 ppm) | | n.a./2.79 |  | | | |
| **Peptide B′-HexNAc (Moxi)** | DPFYTFSIDGHnmTIIEA | +2 | 1145.51355 Da  (-0.99 ppm) | | 32/2.44 |  | | | |
| **Peptide C-HexNAc** | DnVTIRFVT | +2 | 1112.06030 Da  (-0.32 ppm) | | 13/1.61 |  | | | |
| **Peptide C′′-HexNAc** | DVVSTGTPAAGDnVTIRFVT | +2 | 634.32941 Da  (-1.08 ppm) | | 73/4.17 |  | | | |

The three *N*-glycosylation sites are included in the Peptides A-C, respectively.

Ion Score: the main score from Mascot

XCorr: the main score from SEQUEST

N->D: Asn deamidated to Asp (^18^O)

Moxi: Methionine oxidation

n.a.: not available

**Peptide A (N->D)** : LNVIDELTnHTMLK


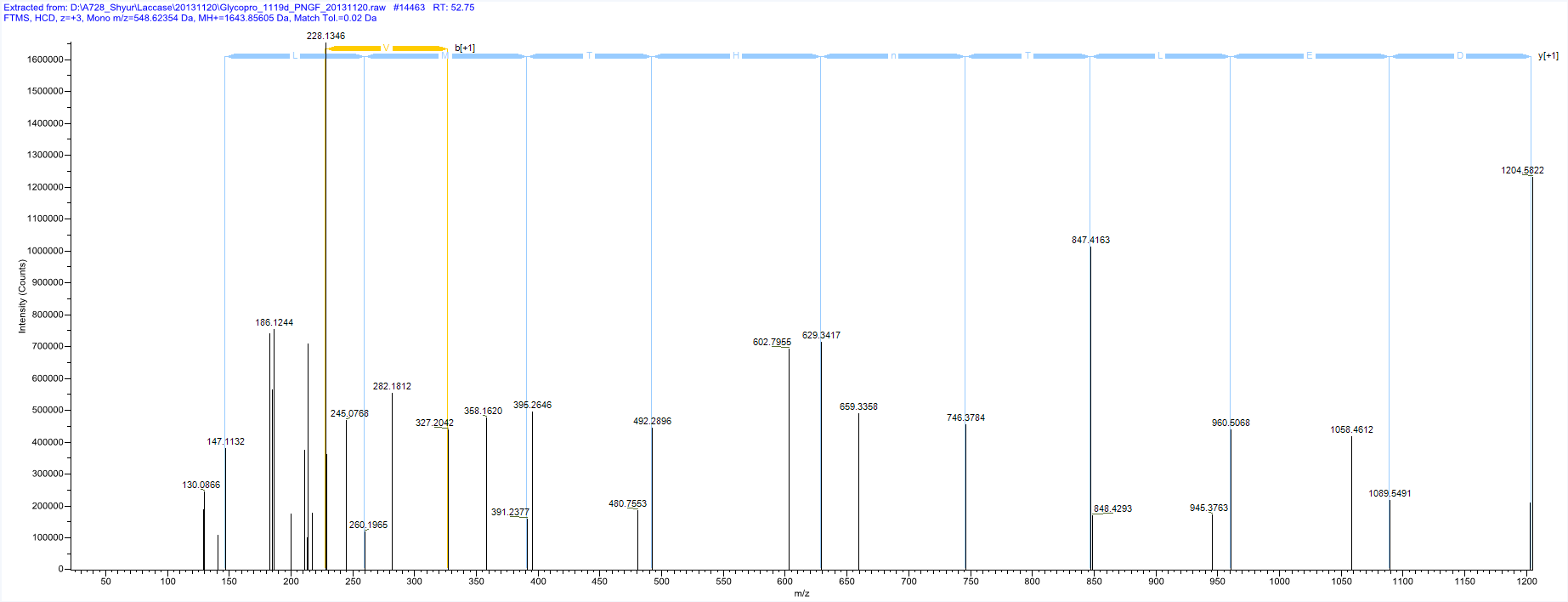


**Peptide A (N->D, Moxi) :** LNVIDELTnHTmLK


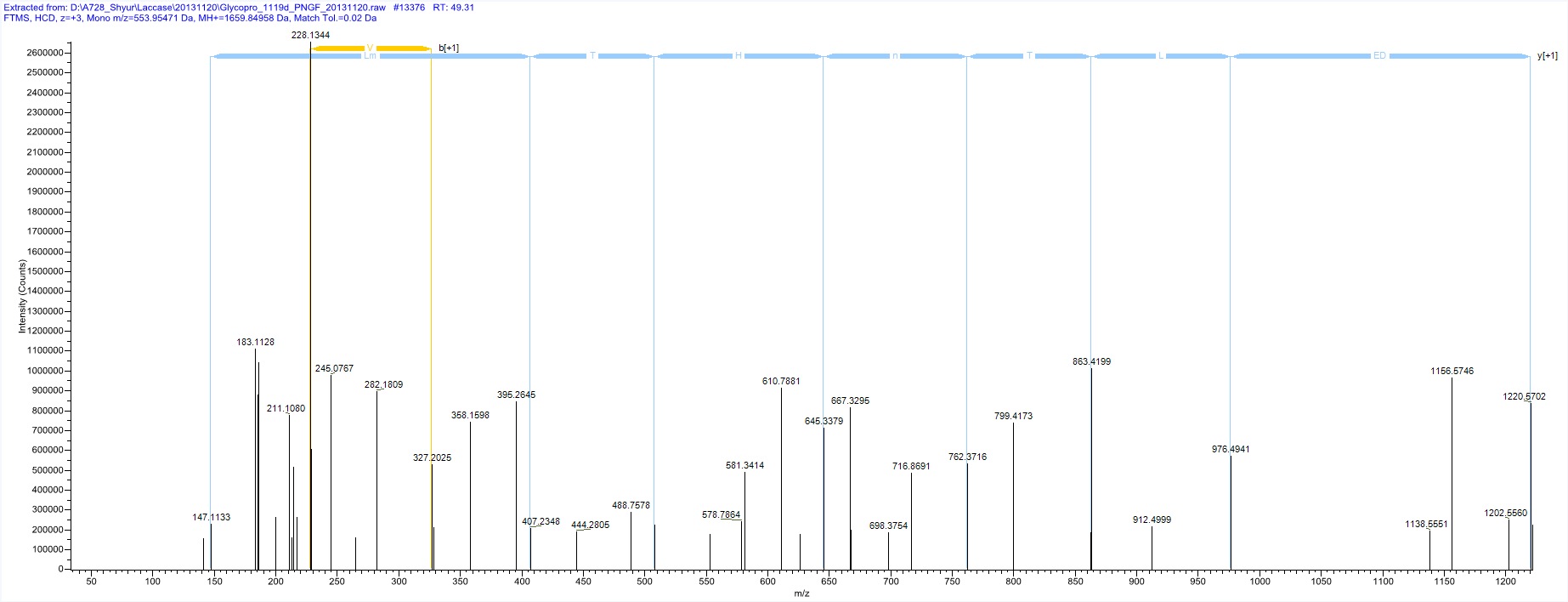


**Peptide C′ (N->D):** DVVSTGTPAAGDnVTIR


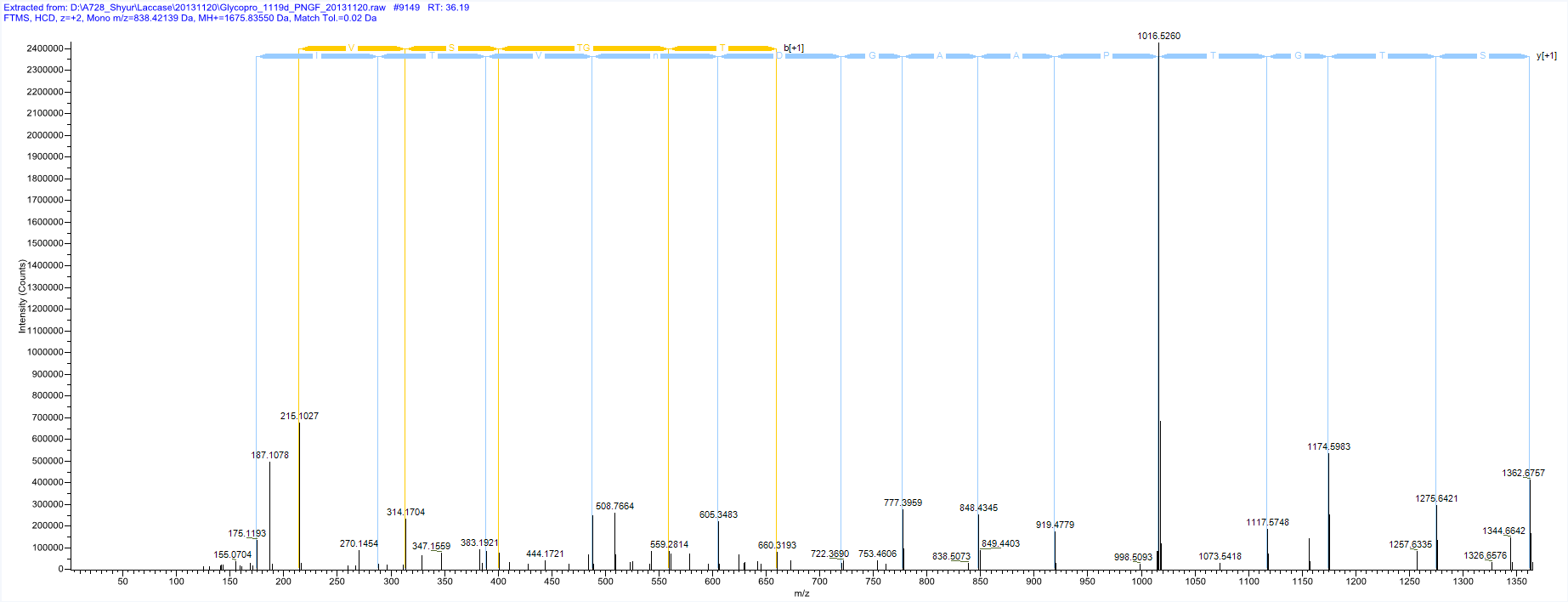


**Peptide B (N->D, Moxi) :** DGHnmTIIEA


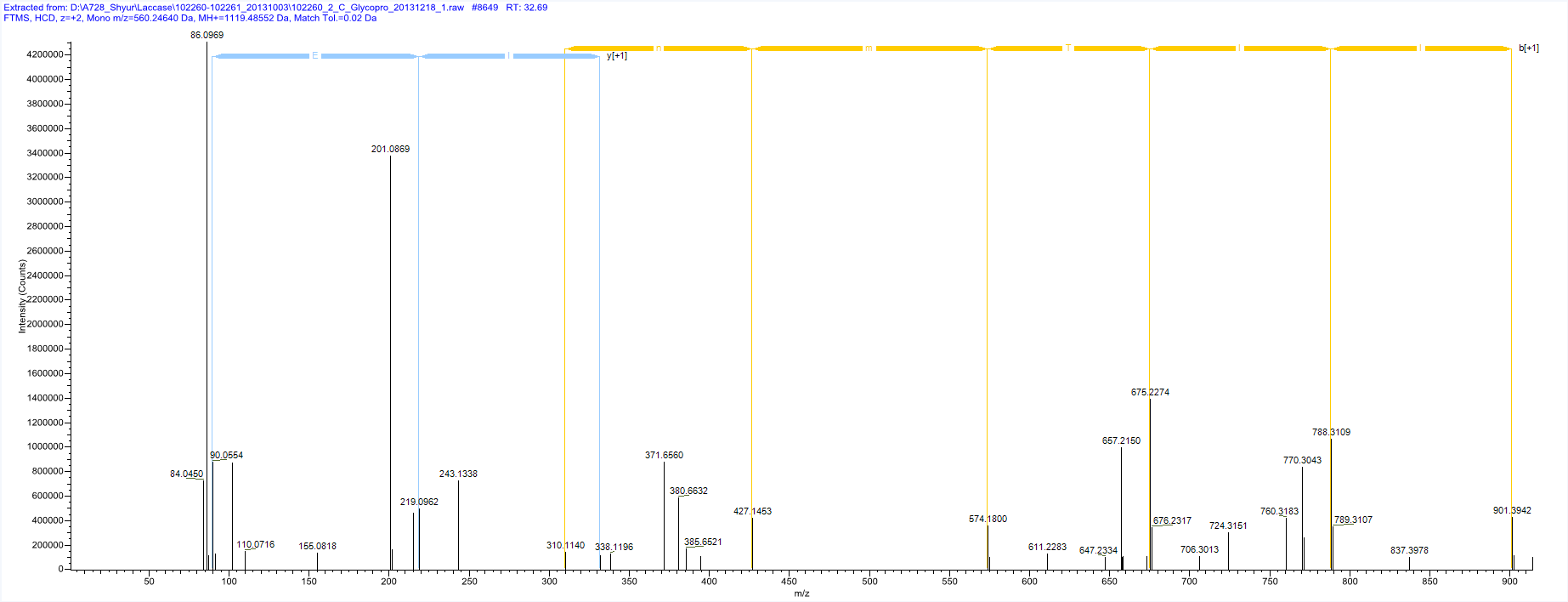


**Peptide C (N->D) :** DnVTIRFVT


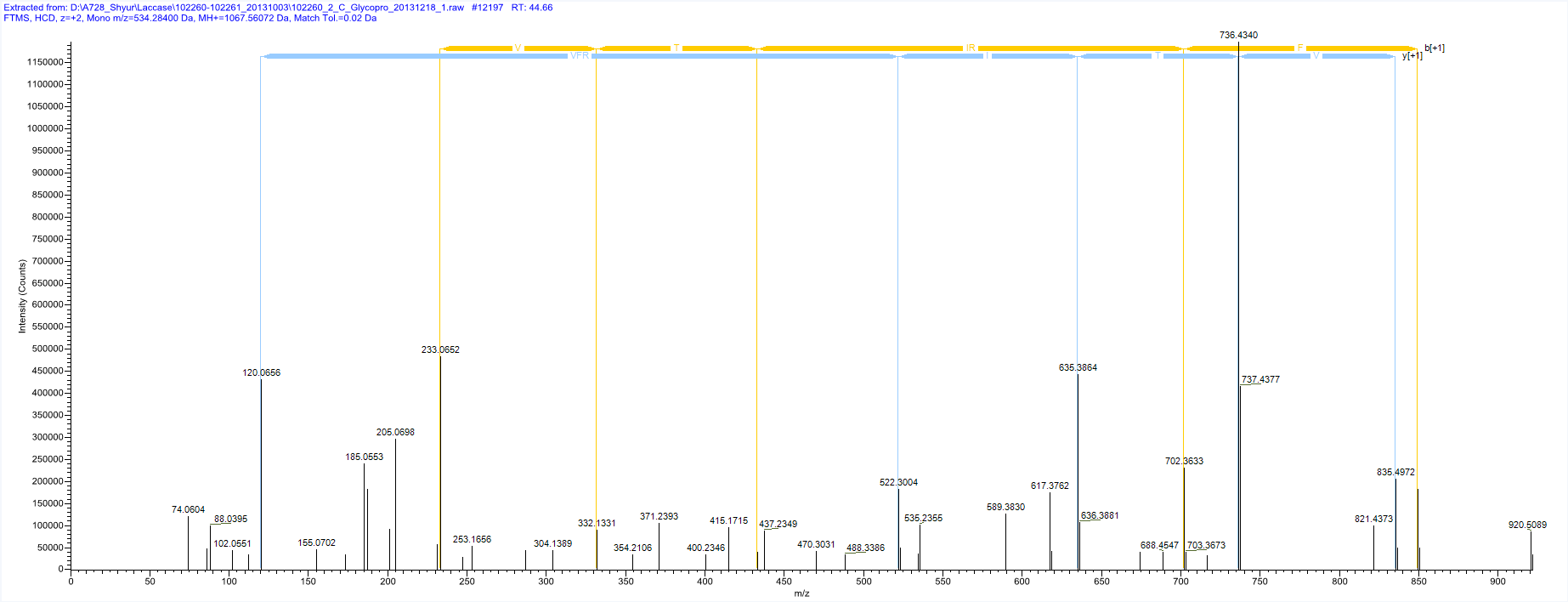


**Peptide A-HexNAc :** LNVIDELTnHTMLK


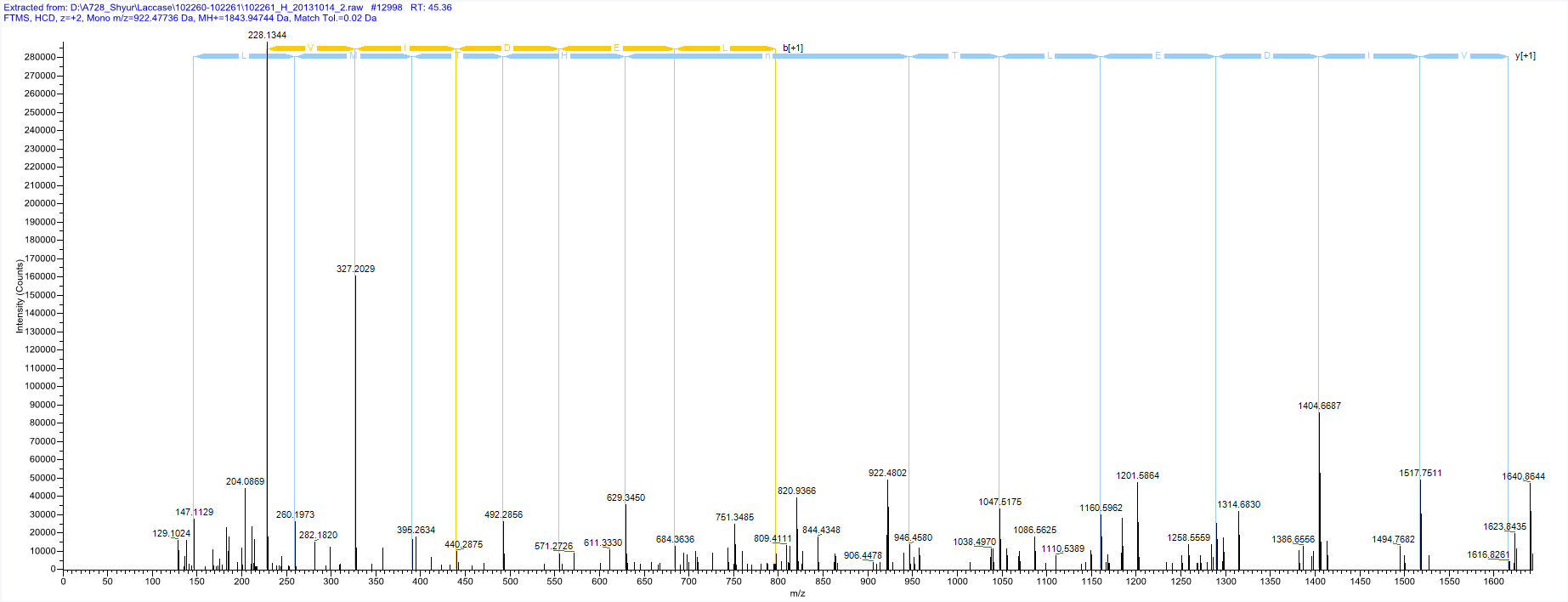


**Peptide A-HexNAc (Moxi) :** LNVIDELTnHTmLK


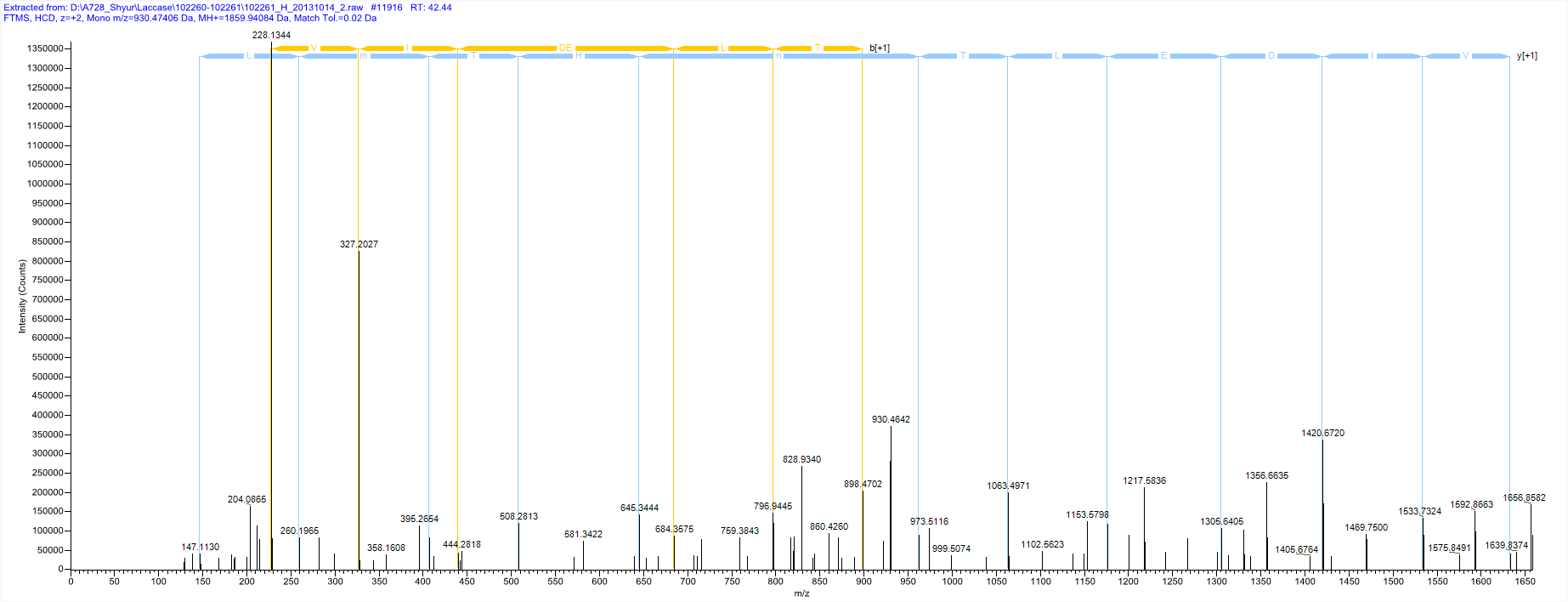


**Peptide C′-HexNAc:** DVVSTGTPAAGDnVTIR


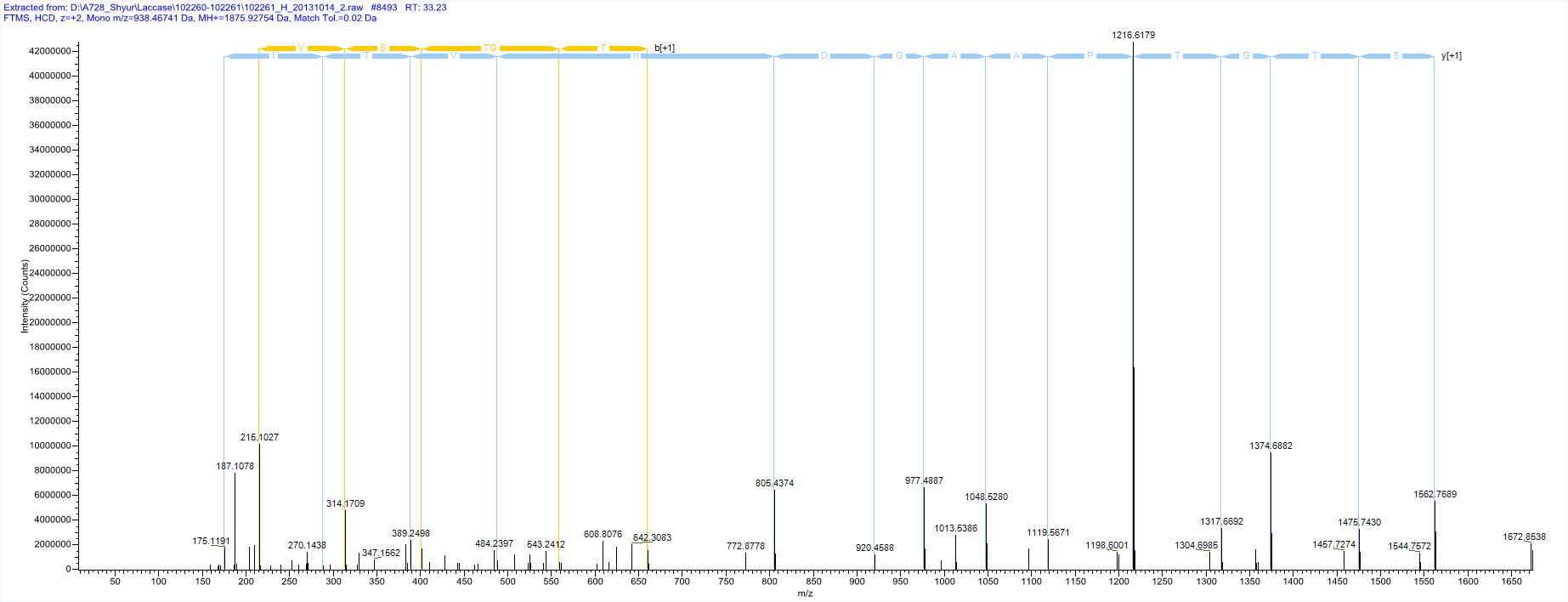


**Peptide B′-HexNAc :** DPFYTFSIDGHnMTIIEA


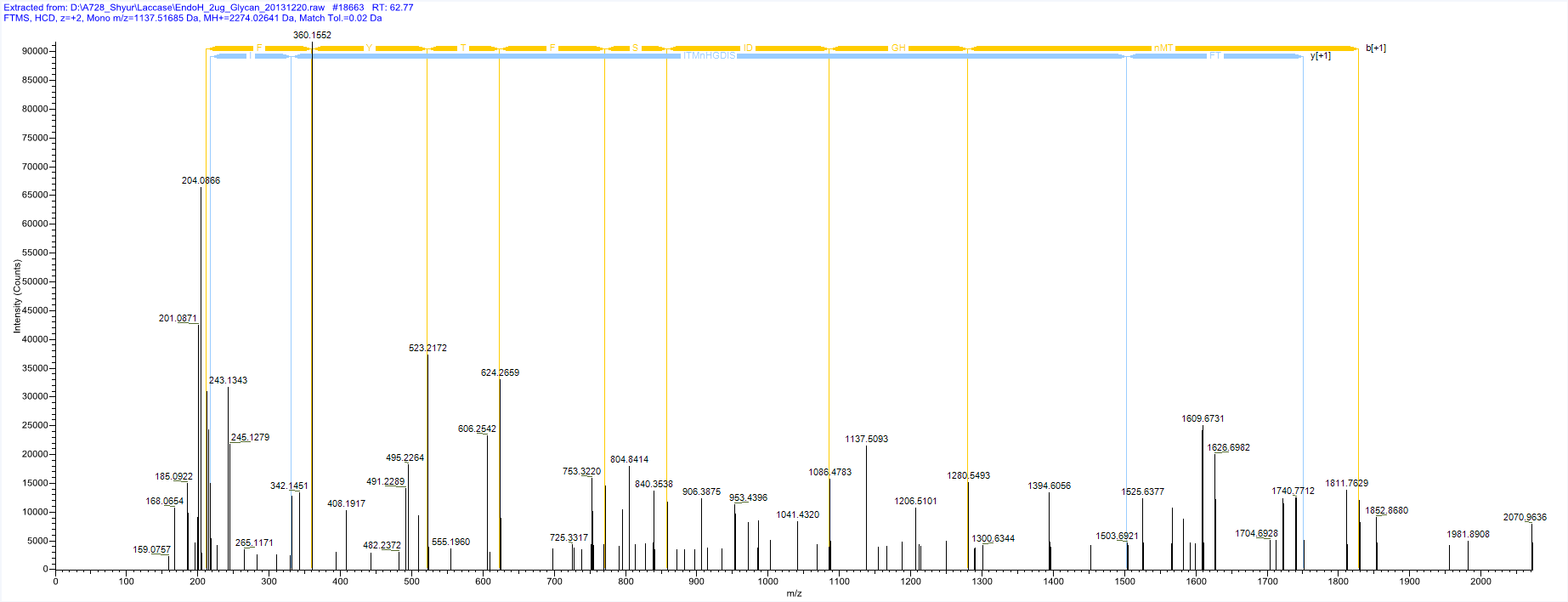


**Peptide B′-HexNAc (Moxi) :** DPFYTFSIDGHnmTIIEA


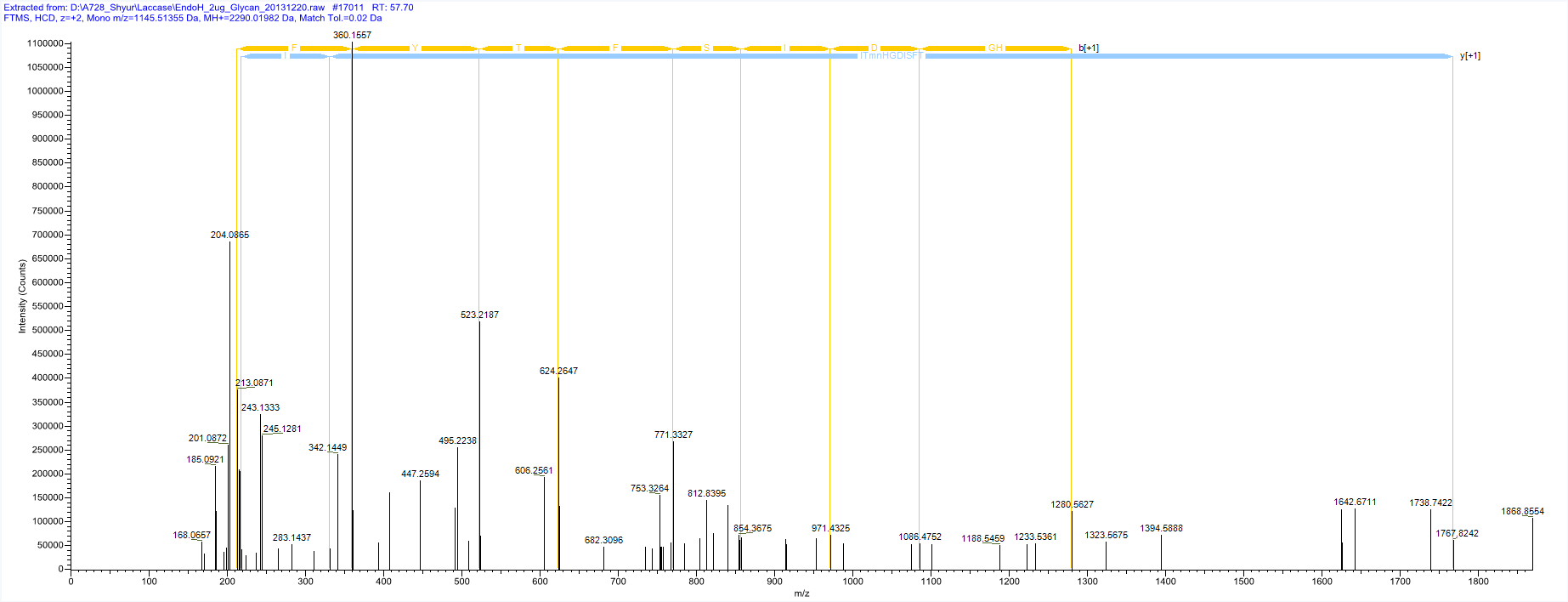


**Peptide C-HexNAc :** DnVTIRFVT


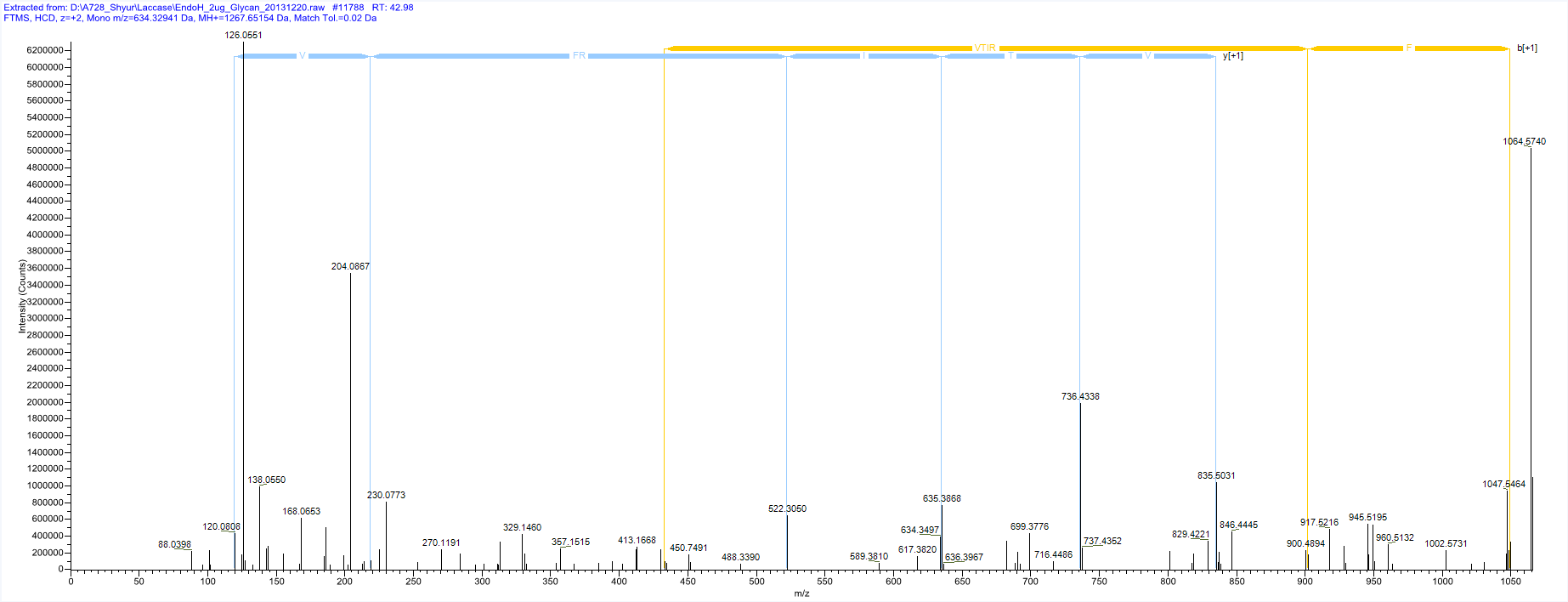


**Peptide C′′-HexNAc :** DVVSTGTPAAGDnVTIRFVT

**
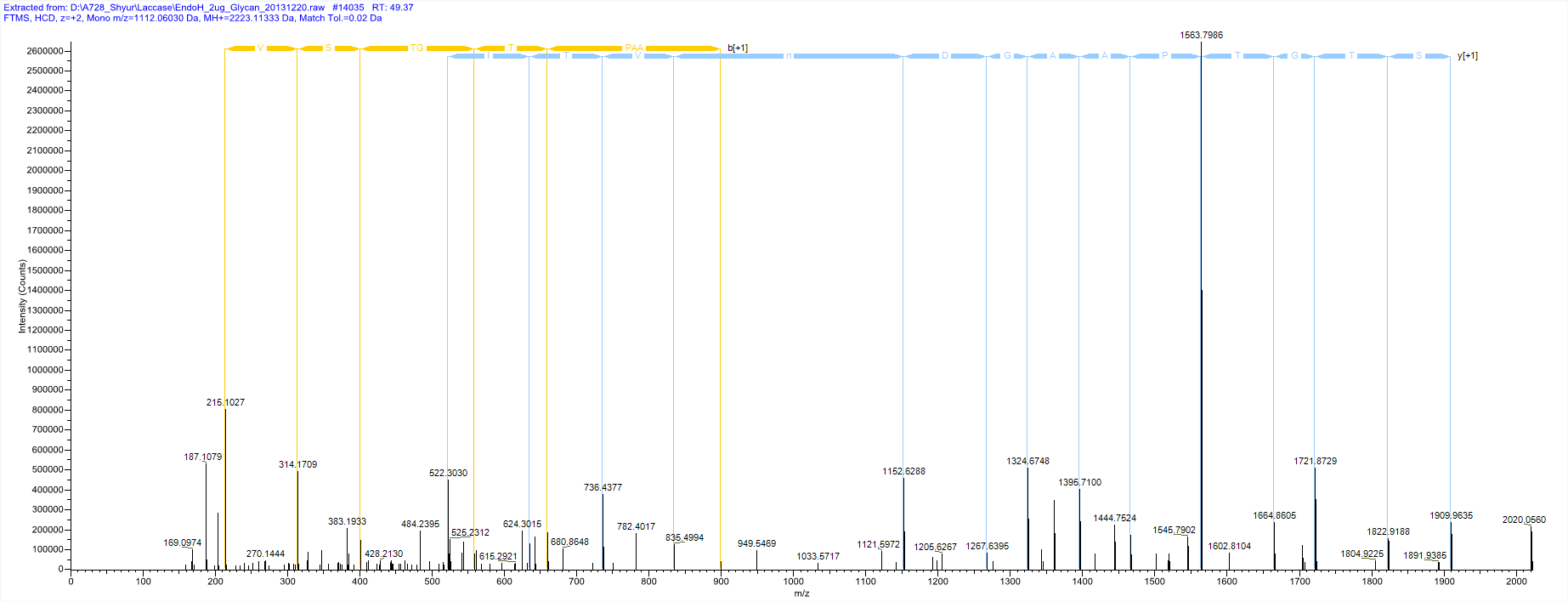
**

**Table B**

**Comparison between the D2-D3 loop-glycan 238 regions of homologous laccases of known structure.***

| **Organism of origin** | **PDB ID** | **Glycosylation status** | **% of hydrophobic amino acids** |
| --- | --- | --- | --- |
| *Trametes versicolor* | 1GYC | yes | 48 |
| *Cerrena maxima* | 2H5U | yes | 52 |
| *Melanocarpus albomyces* | 2Q9O | yes | 50 |
| *Tramete hirsuta* | 3FPX | yes | 62 |
| *Tramete sp.* AH28-2 | 3KW7 | yes | 42 |
| *Trametes trogii* | 2HRG | no | 43 |
| *Coriolus zonatus* | 2HZH | no | 75 |
| *Lentinus tigrinus* | 2QT6 | no | 50 |
| *Pycnoporus cinnabarinus* | 2XYB | no | 48 |
| *Steccherinum ochraceum* | 3T6V | no | 42 |
| *Coriolopsis gallica* | 4A2E | no | 50 |
| *Coriolopsis caperata* | 4JHV | no | 40 |
| *Melanocarpus albomyces* | 1GW0 | alternative | 57 |
| *Botrytis aclada* | 3SQR | alternative | 50 |
| *Botrytis aclada* | 3V9E | alternative | 55 |
| *Lentinus sp.*  (nLcc4, this study) | 3WLG | yes | 78 |

*: proteins are identified by organism of origin and PDB code. Their glycosylation status at glycan 238 is given next, with yes indicating glycosylation at Asn^238^, no stands for no glycosylation whatsoever in the region. Alternative glycosylation indicates that, although position 238 is not glycosylated, a nearby glycosylation site is present, which might have the same function as Asn^238^. Finally, the percentage of hydrophobic amino-acids for the D2-D3 region in direct vicinity of glycan 238 (amino acids 302 to 323) is given for each of the laccases.

**Table C**

**Calculated percentage of protonation**

**at the corresponding pH**

| **Sites** | **pKa*** | **reaction condition**  **pH 2.5** |
| --- | --- | --- |
| Asp^75^ | 3.3 | 86% |
| Asp^162^ | 3.1 | 80% |
| Asp^238^ | 3.9 | 96% |
| Asp^458^ | 4.4 | 99% |

*pKa was calculated by the H++ server [1], while

percentages of protonation were calculated via

the Henderson Hasselbach equation.

**Supplemental Figure Legends**

**Figure A. Asn to Asp mutant MD simulation.** The micro-environments surrounding each of the glycosylation sites are colored as follows: aspartate (Asp) mutants are colored by B-factor, de-glycosylated wild-type is colored grey, and wild-type fully glycosylated nLcc4 is colored yellow. A. N/D 458, B. N/D 238, C. N/D 75, and D. N/D 162. The MD simulation parameters are set as shown in Figure 7 but without the background adjustment.

**Figure B. Simulation model of hypothetical glycosylated rLcc4 protein expressed in the *Pichia* host cell system.** In *Pichia*, it has been suggested that proteins are hyperglycosylated with mannose-rich *N*-glycans (on average with Mannoses 9-14) [2]. Left side: hyperglycosylated rLcc4 modeled with 14 mannoses (Man 14), colored by simulated B-factor. Right side: Simulated B-factor values for the copper ions and SBPLs for rLcc4 under native (GlcNAc_2_Man_5_) and *Pichia* glycosylation (GlcNAc_2_Man_14_). *Pichia* glycosylation consistently reduces plasticity around the binding pocket.

**Figure A**

**Figure B**

**
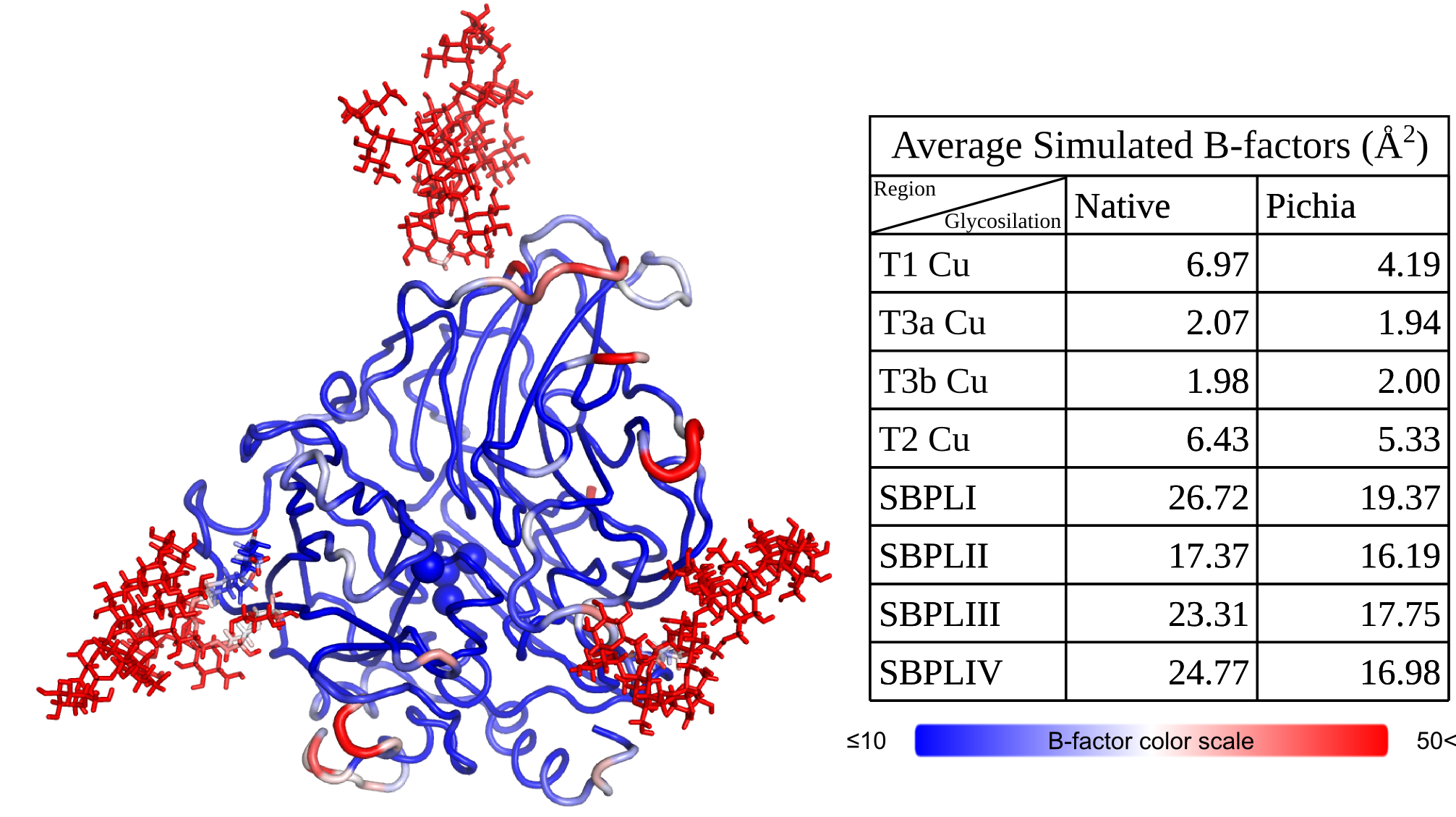
**

**References**

[1] Gordon JC, Myers JB, Folta T, Shoja V, Heath LS, Onufriey A (2005) H++: a server for estimating pK_a_s and adding missing hydrogens to macromolecules. Nucl Acids Res 33: W368–W371.

[2] Hamilton SR, Gerngross TU (2007) Glycosylation engineering in yeast: the advent of fully humanized yeast. Curr Opin Biotechnol 18: 387–392.
